# Discovery and characterization of *tmexCD3-toprJ1* on a plasmid from *Pseudomonas putida* isolated in a public trash can

**DOI:** 10.1128/spectrum.00395-24

**Published:** 2024-08-20

**Authors:** Xiaoqian Long, Jie Li, Hua Yang, Yuehua Gao, Xiaoqun Zeng, Biao Tang

**Affiliations:** 1 State Key Laboratory for Managing Biotic and Chemical Threats to the Quality and Safety of Agro-Products, College of Food Science and Engineering, Ningbo University, Ningbo, China; 2 College of Life Science, Liaocheng University, Liaocheng, China; 3 State Key Laboratory for Managing Biotic and Chemical Threats to the Quality and Safety of Agro-Products & Institute of Agro-Product Safety and Nutrition, Zhejiang Academy of Agricultural Sciences, Hangzhou, Zhejiang, China; 4 Key Laboratory of Systems Health Science of Zhejiang Province, School of Life Science, Hangzhou Institute for Advanced Study, University of Chinese Academy of Sciences, Hangzhou, China; University of Saskatchewan, Saskatoon, Saskatchewan, Canada

**Keywords:** *tmexCD3-toprJ1*, *Pseudomonas putida*, plasmid, environmental

## LETTER

Tigecycline is the last line of defense against bacterial infections, particularly those caused by carbapenem-resistant and polymyxin-resistant bacteria. However, the emerging multidrug-resistant efflux pump gene cluster *tmexCD-toprJ*, which belongs to the resistance–nodulation–division (RND) family, has become a significant concern due to its robust resistance against various antibiotics (1, 2). *tmexCD-toprJ* has been transmitted in a variety of clinical pathogens, among which *tmexCD1-toprJ1*, *tmexCD2-toprJ1*, and *tmexCD4-toprJ4* are mainly detected in *Klebsiella*, and *tmexCD3-toprJ1* and *tmexCD6-toprJ1* are mainly carried in *Pseudomonas* (3–5). *Pseudomonas* is one of the major pathogens of hospital-acquired infections, and because of its high antimicrobial resistance (AMR), treatment is often very difficult and can lead to infection in other patients. At present, there are many studies on *Pseudomonas* carrying *tmexCD-toprJ* gene clusters mainly from clinical samples but relatively few studies on its source in the environment.

**Fig 1:**
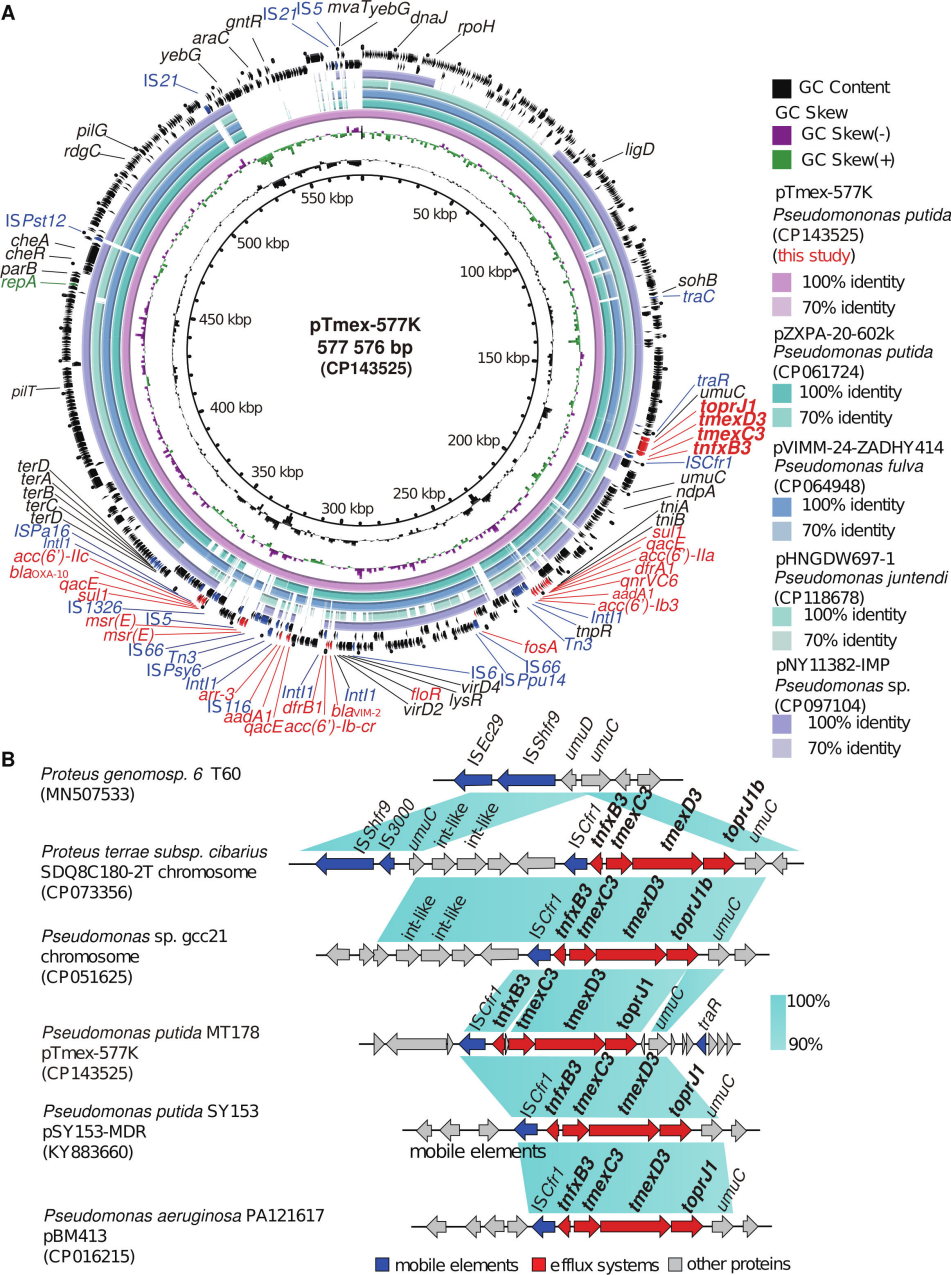
Sequence alignment of plasmid pTmex-577K and the gene environment of *temxCD3-toprJ1* gene cluster. (**A**) Comparative analysis of the *temxCD3-toprJ1* harbored pTmex-577K with other identical plasmids pZXPA-20-602k, pVIM-24-ZDHY414, pNY11382-IMP, and pHNGDW697-1. The red arrow indicates the AMR genes and efflux system, the blue arrow indicates mobile elements, the green arrow indicates the plasmid replication, and the gray and black arrow indicates other proteins. (**B**) Comparison of the genetic context of *temxCD3-toprJ1* between *P. terrae* subsp. cibarius SDQ8C180-2T, *Pseudomonas* sp. gcc21, *P. aeruginosa* PA121617, and *P. putida* SY153.

In 2023, a total of 178 samples were collected from garbage cans in Liaocheng, Shandong Province, and strain MT178 was subsequently isolated using a Luria–Bertani agar plate supplemented with 2 mg/L tigecycline. Meanwhile, a PCR screening assay confirmed that *tmexCD-toprJ* was carried by this strain (Table S1). Finally, the complete genome sequence was obtained by using both Illumina NovaSeq and Oxford Nanopore GridION sequencing platforms, followed by hybrid assembly with Unicycler v0.5.0. The *tmexCD3-toprJ1*, *bla*_VIM-2_, and *bla*_OXA-10_ co-exist on a large plasmid called pTmex-577K (Fig. 1A), and this plasmid was untypable based on PlasmidFinder prediction. According to our previous method of conjugation experiments (6), it was found that this plasmid demonstrates the capability of undergoing conjugative transfer to *Pseudomonas aeruginosa* PAO1 (Fig. S1) at a transfer frequency of (2.64 ± 0.11) × 10^−5^, while no such transfer was observed in *Escherichia coli* J53. The strain exhibited resistance to tigecycline with a minimum inhibitory concentration of 2 mg/L and harbored multiple regions conferring resistance (Table S2). BLASTn analysis revealed that the backbone sequence of pTmex-577K shared high similarity (>85% coverage rate and >99.55% homology) with untypable plasmids such as pZXPA-20-602k, pVIEM-24-ZDHY414, pNY11382-IMP, and pHNGDW697-1, which are predominantly found in animal feces or clinical samples. The presence of *tmexCD3-toprJ1* was observed in all four plasmids (Fig. 1A). The genetic environment of the gene cluster was further studied, and the *tmexCD3-toprJ* genetic structure of IS*Cfr1-tnfxB3-hp-tmexCD3-toprJ1-hp-umuC* was found to be similar to strains *Pseudomonas putida* SY153 and *Pseudomonas aeruginosa* PA121617. It is important to note that this *tmexCD3-toprJ1* differs from the previously discovered *temxCD3-toprJ1*, as it is located near the *umuC* gene instead of being inserted into the *umuC*-like as observed in the fragment carried by the *Proteus terrae* subsp. cibarius strain SDQ8C180-2T, and two integrase genes were found upstream of *temxCD3-toprJ1* (Fig. 1B) (7). Furthermore, phylogenetic analysis indicated that strain MT178 was closely related to *P. putida* strains SY153 carrying *tmexCD1-toprJ1* and 12969 carrying an unclassified type of *tmexCD-toprJ*, which were isolated from clinical samples in China in 2012 and 2015, respectively (Fig. S2) (Table S3).

The *tmexCD-toprJ* variants, ranging from *tmexCD1-toprJ1* to *tmexCD6-toprJ1*, have been widely detected in clinical, animal, and environmental settings (1, 3, 4, 8, 9). Currently, *tmexCD1-toprJ1* to *tmexCD3-toprJ1* (1, 8) and *tmexCD6-toprJ1* (3) can be detected in *Pseudomonas* isolates from hospital and environmental samples. However, comprehensive global-scale genomic analysis has only been conducted for *P. aeruginosa* and *P. putida* species (3). Therefore, to gain insights into the worldwide distribution and prevalence of *Pseudomonas* species harboring the *tmexCD-toprJ* gene cluster, we mined all *Pseudomonas* isolates carrying these gene clusters from the GenBank database from 1993 to 2023, and a total of 571 *Pseudomonas* strains were identified. Among these, *P. aeruginosa* exhibited the highest detection rate at 71.84% (*n* = 410) (Fig. S3). These strains were primarily isolated from China and other Asian regions (Fig. S4). The primary sources of isolation for *Pseudomonas* strains carrying the *tmexCD-toprJ* gene were predominantly human (71.25% of cases, *n* = 407), followed by unknown (*n* = 70), environmental (*n* = 66), and animal (*n* = 28) origins (Fig. S5A). This observation may be attributed to the higher usage of antibiotics in hospitals compared to other sources. Notably, the abundance of *tmexCD-toprJ* was found to be higher in unknown sources as compared to animal and environmental origins. The category of unknown sources likely encompasses individuals, animals, and the environment, with a significant contribution from human carriers. Furthermore, an overall trend analysis revealed an initial increase followed by a subsequent decline in the number of strains harboring *tmexCD-toprJ* from all origins, reaching its peak during 2018–2020 (Fig. S5B). The unclassified *tmexCD-toprJ* was detected in 57.59% (*n* = 330) of the 571 analyzed *Pseudomonas* strains (Fig. S6A). The highest number of *tmexCD-toprJ* variants occurred between 2018 and 2020 (Fig. S6B). Predominantly, the isolates carried the *tmexCD3-toprJ1* and *tmexCD1-toprJ1*, which were primarily derived from humans (Fig. S6C). Notably, among the 571 *Pseudomonas* strains (Table S3), the *tmexCD-toprJ* co-existed with *bla*_NDM_ in 27 strains and *tet*(X) in 3 strains. However, the co-existence of the *tmexCD-toprJ* and *mcr* in *Pseudomonas* has not been reported (Table S4). The MLST tool (https://github.com/tseemann/mlst) is based on the complete bacterial genome sequence classification, of which 388 strains of *Pseudomonas aeruginosa* and 10 strains of *P. putida* have been identified in 90 species and 15 different sequence type (STs). The most common lineages were *P. aeruginosa* ST235, ST1418, and *P. putida* ST144, and ST235 is undoubtedly the most relevant high-risk clone (Fig. S7) (10). A phylogenetic tree was constructed using Roary based on core genome SNPs. The 410 strains of *P. aeruginosa* mainly formed four different clades, mainly from Chinese clinical samples. The four clades mainly carried *tmexCD3-toprJ1*, and *tmexCD1-toprJ1* and *tmexCD6-toprJ1* could also be detected. Another 13 *Pseudomonas juntendi* and 10 *Pseudomonas chengduensis* are similarly divided into three clades, mainly carrying the undefined *tmexCD-toprJ* variant (Fig. S8 and S9). The identification of *tmexCD-toprJ* implies a significant role of *Pseudomonas* in facilitating the dissemination of antimicrobial resistance genes (ARGs) across temporal and spatial scales. The broad distribution and prolonged persistence highlight the pivotal role of *Pseudomonas* as a key vector for ARG propagation.

Our findings provide a significant contribution to the epidemiology of *tmexCD-toprJ-*positive *Pseudomonas*, while the emergence of multiple variants signifies a further evolutionary and diversifying trend within the gene cluster. This underscores the ongoing emergence and adaptation of antibiotic resistance mechanisms in bacterial populations, thereby emphasizing the necessity for continuous surveillance and research to monitor both the prevalence and characteristics of this novel variant, as well as its potential impact on public health.

## Data Availability

The complete genome sequences of strain MT178 were deposited at GenBank with accession numbers CP143524-CP143525.

## SUPPLEMENTAL MATERIAL

Supplemental material spectrum.00395-24-s0001.pdf Tables S1, S2, S4; Fig. S1 to S9.

Table S3 spectrum.00395-24-s0002.pdf Genomic information for all the pseudomonas strains.

ASM does not own the copyrights to Supplemental Material that may be linked to, or accessed through, an article. The authors have granted ASM a non-exclusive, world-wide license to publish the Supplemental Material files. Please contact the corresponding author directly for reuse.
