# Supplementary material for: Discovery and characterization of tmexCD3-toprJ1 on a plasmid from Pseudomonas putida isolated in a public trash can
Source: Microbiol Spectr. 2024 Aug 20;12(10):e00395-24. doi: 10.1128/spectrum.00395-24 (PMC11448381; doi:10.1128/spectrum.00395-24)
Supplement: Supplemental material — Tables S1, S2, S4; Fig. S1 to S9. [file spectrum.00395-24-s0001.pdf]

Table S1. Primers are used to detect the *tmexCD-toprJ* gene cluster.

**Primer sequences**	**Primer name (5'-3')**	**Size (bp)**
tmexC-F	TTCCGTGATCTCCTGTTTG	880
tmexC-R	GATGGCGTTCTGGTTGAG	
tmexD-F	CAGCCAGGACTACAACTTC	1314
tmexD-R	TAGAGGAACTTCGGATTGC	

Table S2. Results of antimicrobial susceptibility testing and genetic characterization of strain MT178.

**Antibiotics**	**MIC (mg/L)**	**Interpretation**	**ARGs**
ampicillin	>512	NA	*bla*$_{VIM-2}$
amoxicillin/clavulanate	256/128	NA	*bla*$_{OXA-10}$、*bla*$_{VIM-2}$
gentamycin	>512	R	*aac(6')-IIa*、*aac(6')-IIc*
tetracycline	64	R	*tmexCD3-toprJ1*
spectinomycin	>512	NA	*aadA1*
florfenicol	>256	NA	-
sulfisoxazole	>512	R	*sul1*
trimethoprim/sulfamethoxazole	>32/608	R	-
ceftiofur	>256	R	*bla*$_{VIM-2}$
ceftazidime	>256	R	*bla*$_{VIM-2}$
enrofloxacin	>32	NA	-
ofloxacin	>64	R	-
meropenem	16	R	-
colistin	16	NA	-
tigecycline	2	NA	*tmexCD3-toprJ1*
Types of antibiotics not included in this experiment	NA	NA	*aac(6')-Ib-cr*、*aac(6')-Ib3*、*qnrVC6*、*qacE*、*dfrA1*、*dfrB1*、*arr-3*、*mph(E)*、*msr(E)*、

S= susceptibility, R=resistance, I= intermediate; NA, not applicable; -, none.

Table S4. Information regarding the simultaneous presence of *tmexCD-toprJ* with strains carrying resistance genes to carbapenem and tigecycline

**accession numbers**	**Strains**	***tmexC***	***tmexD***	***toprJ***	**Carbapenem**	**Tigecycline**	**Year**	**Hosts**	**Country**
CP035739.1	*P. aeruginosa*	*tmexC6*	*tmexD6*	*toprJ1b*	*bla*$_{NDM-1}$	-	2014	Homo sapiens	Poland
JASGOH010000001.1	*P. juntendi*	*tmexC3*	*tmexD3*	*toprJ1*	*bla*$_{NDM-1}$	-	2015	Homo sapiens	Pakistan
JAACJB010000039.1	*P. aeruginosa*	*tmexC*	*tmexD2*	*toprJ1*	*bla*$_{NDM-1}$	-	2016	Homo sapiens	India
SWEL01000001.1	*Pseudomonas asiatica*	*tmexC3*	*tmexD3*	*toprJ1*	*bla*$_{NDM-1}$	-	2016	Homo sapiens	Myanmar
JASGRV010000002.1	*Pseudomonas mendocina*	*tmexC3*	*tmexD3*	*toprJ1*	*bla*$_{NDM-1}$	-	2016	Homo sapiens	Bangladesh
ABEUSX020000115.1	*P. aeruginosa*	*tmexC*	*tmexD2*	*toprJ1*	*bla*$_{NDM-1}$	-	2017	Homo sapiens	USA
ABEUTB020000069.1	*P. aeruginosa*	*tmexC*	*tmexD2*	*toprJ1*	*bla*$_{NDM-1}$	-	2017	Homo sapiens	USA
ABEUSY020000157.1	*P. aeruginosa*	*tmexC*	*tmexD2*	*toprJ1*	*bla*$_{NDM-1}$	-	2017	Homo sapiens	USA
SWEH01000003.1	*P. asiatica*	*tmexC*	*tmexD*	*toprJ*	*bla*$_{NDM-1}$	-	2017	Homo sapiens	Myanmar
ABEUSV020000058.1	*P. aeruginosa*	*tmexC*	*tmexD2*	*toprJ1*	*bla*$_{NDM-1}$	-	2018	Homo sapiens	USA
DAHNSC010000156.1	*P. aeruginosa*	*tmexC3*	*tmexD3*	*toprJ1*	*bla*$_{NDM-1}$	-	2019	Homo sapiens	Australia
DAJRUK010000184.1	*P. aeruginosa*	*tmexC*	*tmexD3*	*toprJ*	*bla*$_{NDM-1}$	-	2019	Homo sapiens	Australia
DAFPEV010000205.1	*P. aeruginosa*	*tmexC3*	*tmexD3*	*toprJ1*	*bla*$_{NDM-1}$	-	2019	Homo sapiens	Australia
ABEURG020000205.1	*P. aeruginosa*	*tmexC*	*tmexD*	*toprJ*	*bla*$_{NDM-1}$	-	2020	Homo sapiens	USA
ABKQLC020000122.1	*P. aeruginosa*	*tmexC1*	*tmexD2*	*toprJ1*	*bla*$_{NDM-1}$	-	2020	Homo sapiens	USA
JASUWM010000002.1	*P. aeruginosa*	*tmexC3*	*tmexD3*	*toprJ1*	*bla*$_{NDM-1}$	-	2020	Homo sapiens	Australia
JASUWJ010000002.1	*P. aeruginosa*	*tmexC3*	*tmexD3*	*toprJ1*	*bla*$_{NDM-1}$	-	2020	Homo sapiens	Australia
JASUWC010000002.1	*P. aeruginosa*	*tmexC3*	*tmexD3*	*toprJ1*	*bla*$_{NDM-1}$	-	2020	Homo sapiens	Australia
DAFPGV010000001.1	*P. aeruginosa*	*tmexC3*	*tmexD3*	*toprJ1*	*bla*$_{NDM-1}$	-	2020	Homo sapiens	Australia
CP091088.1	*P. juntendi*	*tmexC*	*tmexD*	*toprJ1*	*bla*$_{NDM-1}$	-	2021	Homo sapiens	China
JAJTOR010000034.1	*P. juntendi*	*tmexC*	*tmexD*	*toprJ1*	*bla*$_{NDM-1}$	-	2021	Homo sapiens	China

ABKGII020000029.1	*P. aeruginosa*	*tmexC1*	*tmexD2*	*toprJ1*	*bla*$_{NDM-1}$	-	2022	Homo sapiens	USA
ABKQLE020000283.1	*P. aeruginosa*	*tmexC1*	*tmexD2*	*toprJ1*	*bla*$_{NDM-1}$	-	2022	Homo sapiens	USA
ABNXCN010000107.1	*P. aeruginosa*	*tmexC3*	*tmexD*	*toprJ1*	*bla*$_{NDM-1}$	-	2022	Homo sapiens	USA
CP137486.1	*P. aeruginosa*	*tmexC*	*tmexD*	*toprJ*	*bla*$_{NDM-1}$	-	2022	Homo sapiens	South Korea
CP035739.1	*P. aeruginosa*	*tmexC6*	*tmexD6*	*toprJ1b*	*bla*$_{NDM-1}$	-	2014	Homo sapiens	Poland
JASGOH010000001.1	*P. juntendi*	*tmexC3*	*tmexD3*	*toprJ1*	*bla*$_{NDM-1}$	-	2015	Homo sapiens	Pakistan
JACAKF010000002.1	*P. aeruginosa*	*tmexC*	*tmexD3*	*toprJ1*	-	*tet*(X6)	2019	Animals	China
JACAKE010000002.1	*P. aeruginosa*	*tmexC*	*tmexD3*	*toprJ1*	-	*tet*(X6)	2019	Animals	China
JAKHEW010000003.1	*P. aeruginosa*	*tmexC3*	*tmexD3*	*toprJ1*	-	*tet*(X5)	2019	Animals	China

“-”, no data

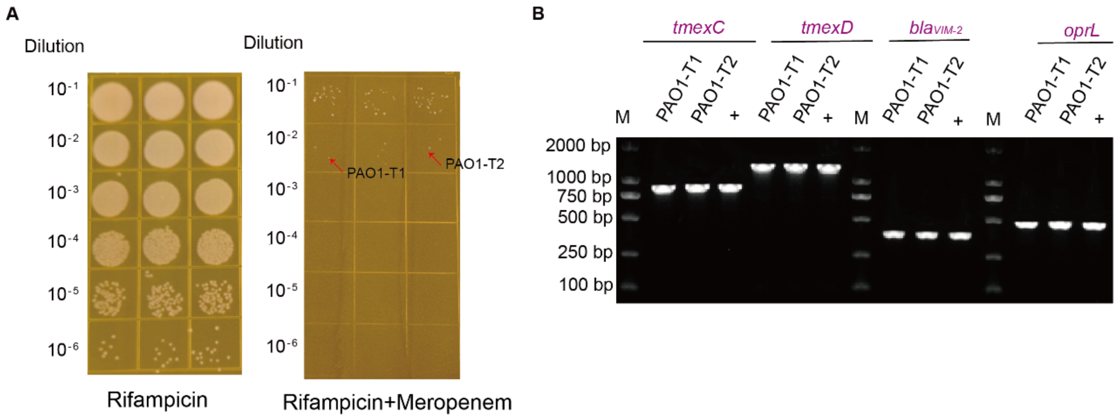

Figure S1. (A) Conjugation transfer test of *P. putida* MT178 with rifampicin-resistant *Pseudomonas aeruginosa* PAO1 served as the recipient strain. From left to right are the screening results under the selective pressure of rifampicin (50 mg/L), rifampicin (50 mg/L) and meropenem (8 mg/L). The red arrow represents the transconjugants. (B) PCR verification of transconjugants. “+”: positive. *tmexC* and *tmexD* were identified as part of the *tmexCD3-toprJ1* cluster, while *oprL* gene served as a specific primer for *P. aeruginosa*.

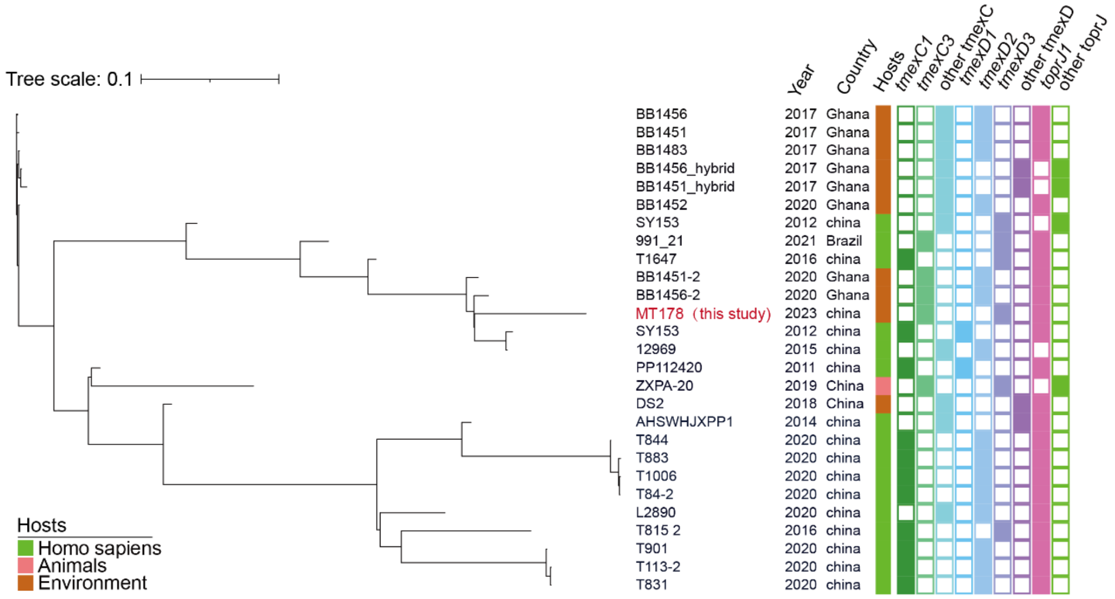

Figure S2. Phylogenetic analysis of *P. putida* carrying the *tmexCD-toprJ* gene cluster. The metadata of different isolates are arranged from left to right: year, country, hosts, and *tmexCD-toprJ* variant type. The red font indicates the strains studied in this study.

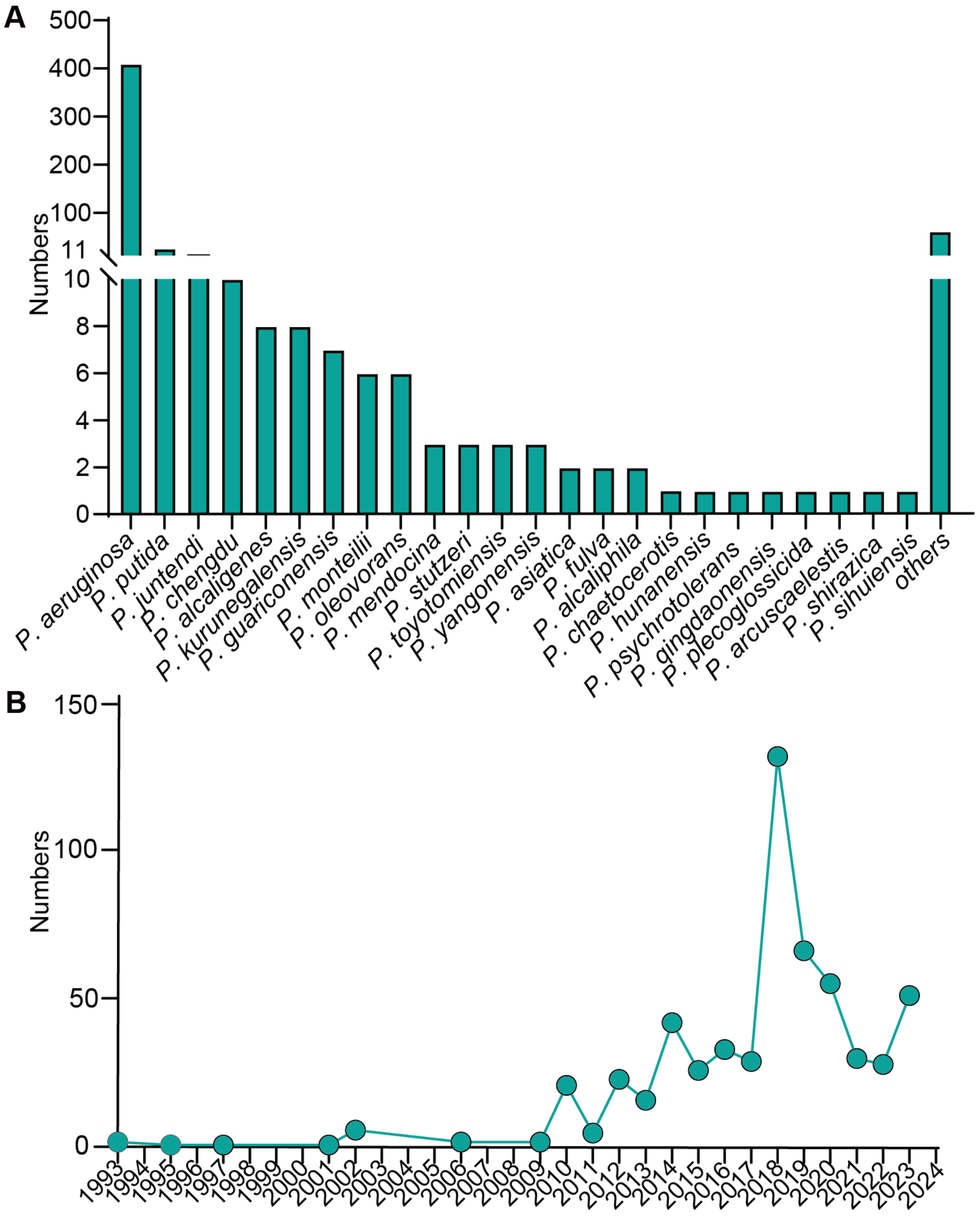

Figure S3. Species distribution of 571 *Pseudomonas* strains and its change over time. (A) 571 strains of *Pseudomonas* belong to the distribution of different *Pseudomonas* species; (B) Global trends in *Pseudomonas* strains carrying *tmexCD-toprJ* from 1993 to 2023.

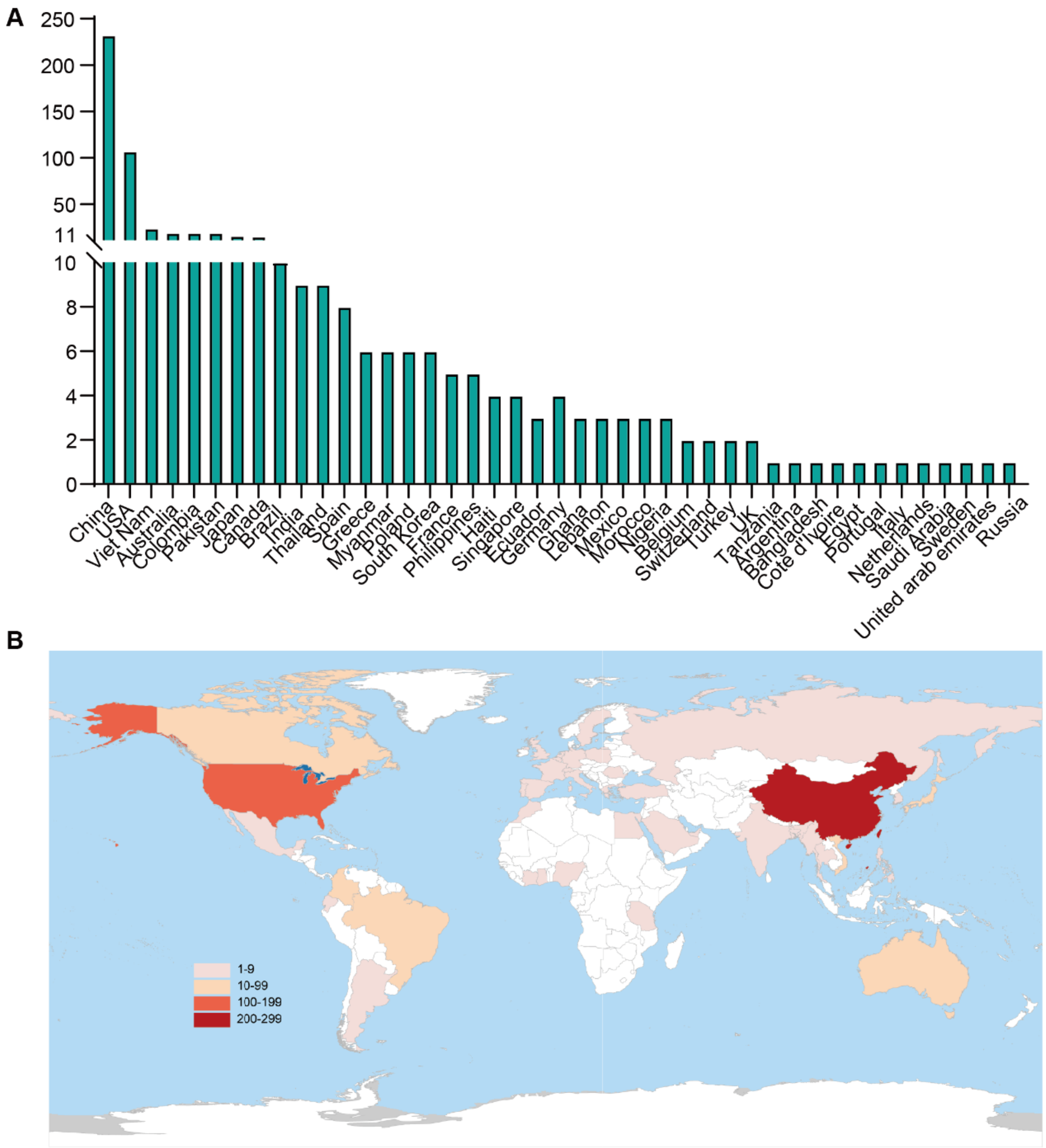

Figure S4. Regional distribution of 571 *Pseudomonas* strains. (A) Distribution of 571 of *Pseudomonas* strains in different countries; (B) Map distribution of 571 *Pseudomonas* strains in different countries.

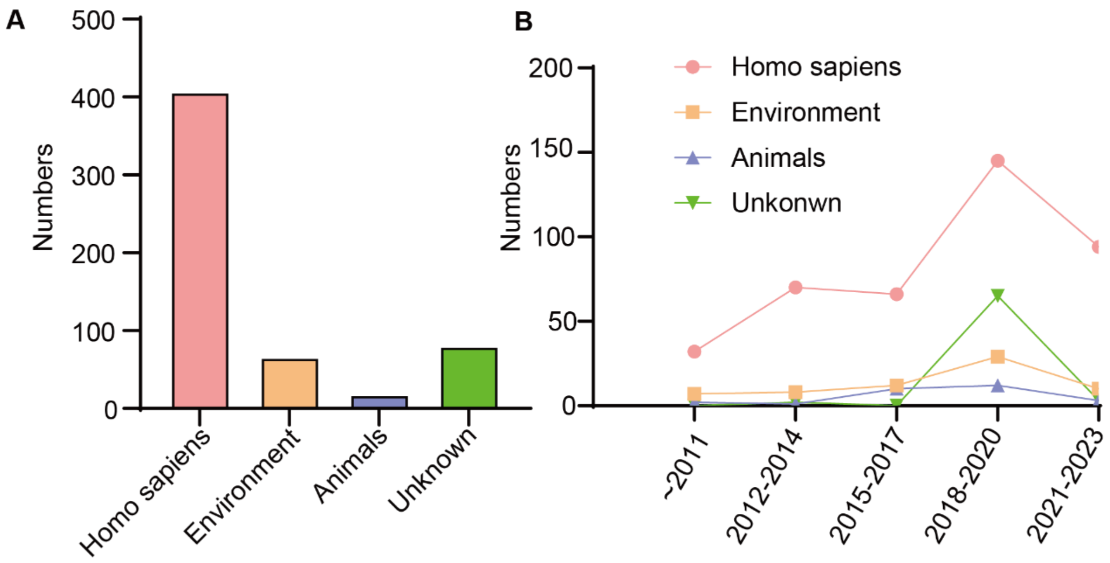

Figure S5. Origin characteristics of 571 strains of *Pseudomonas*. (A) Distribution of 571 *Pseudomonas* strains from different hosts; (B) Isolation of 571 *Pseudomonas* strains from different hosts over time.

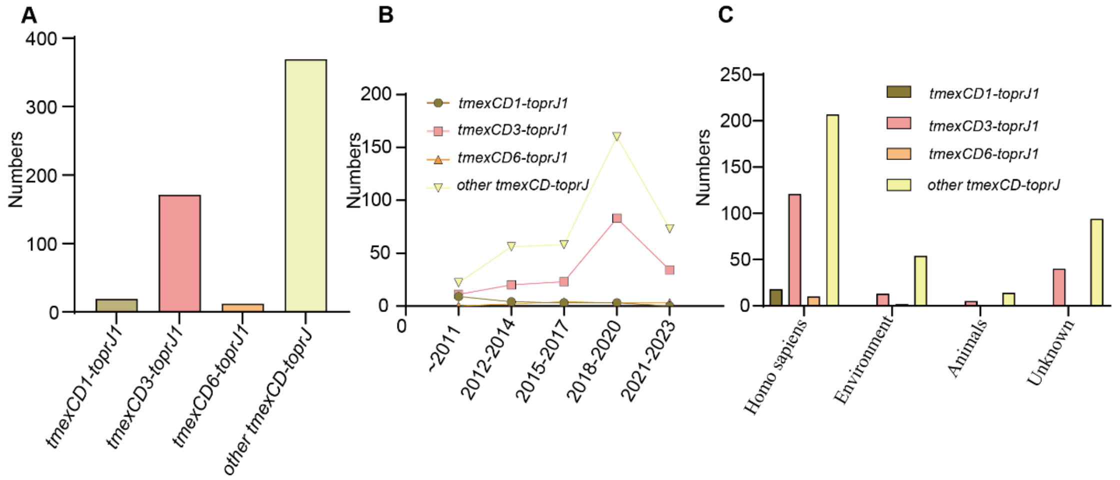

Figure S6. Characteristics of *tmexCD-toprJ* variants of 571 *Pseudomonas* strains. (A) Distribution of *tmexCD1-toprJ1*, *tmexCD3-toprJ1, tmexCD6-toprJ1* and other *tmexCD-toprJ* carried by 571 *Pseudomonas* strains; (B) The number of 571 *Pseudomonas* strains carrying *tmexCD1-toprJ1, tmexCD3-toprJ1, tmexCD6-toprJ1* and other *tmexCD-toprJ* changed in different periods; (C) 571 strains of *Pseudomonas* carrying *tmexCD1-toprJ1*, *tmexCD3-toprJ1, tmexCD6-toprJ1* and other *tmexCD-toprJ* in different hosts.

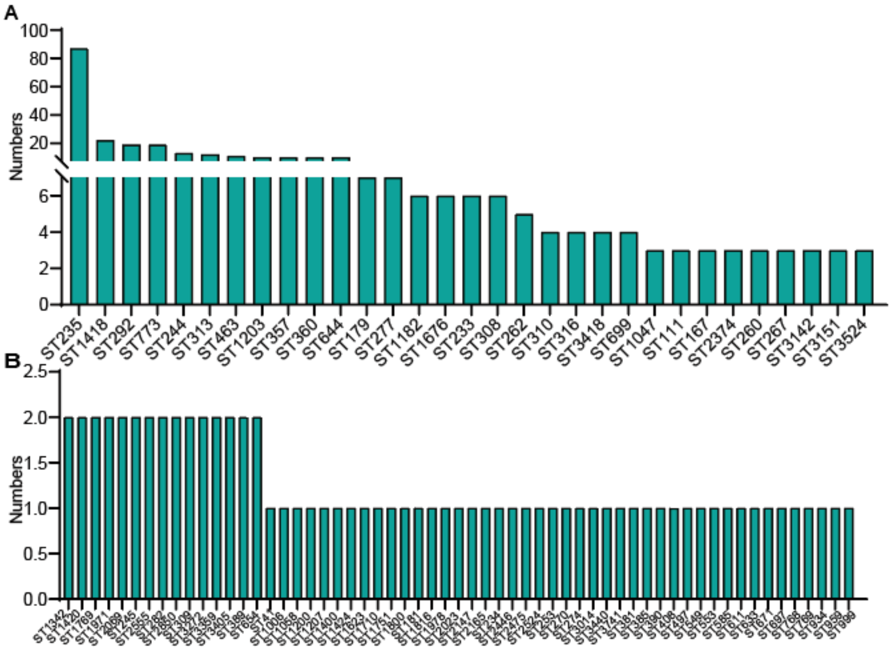

Figure S7. Distribution of 383 *P. aeruginosa* strains identified as MLST. (A) Distribution of 3 or more *P. aeruginosa* strains carrying the same ST type; (B) Distribution of a *P. aeruginosa* strains carrying the same ST type.

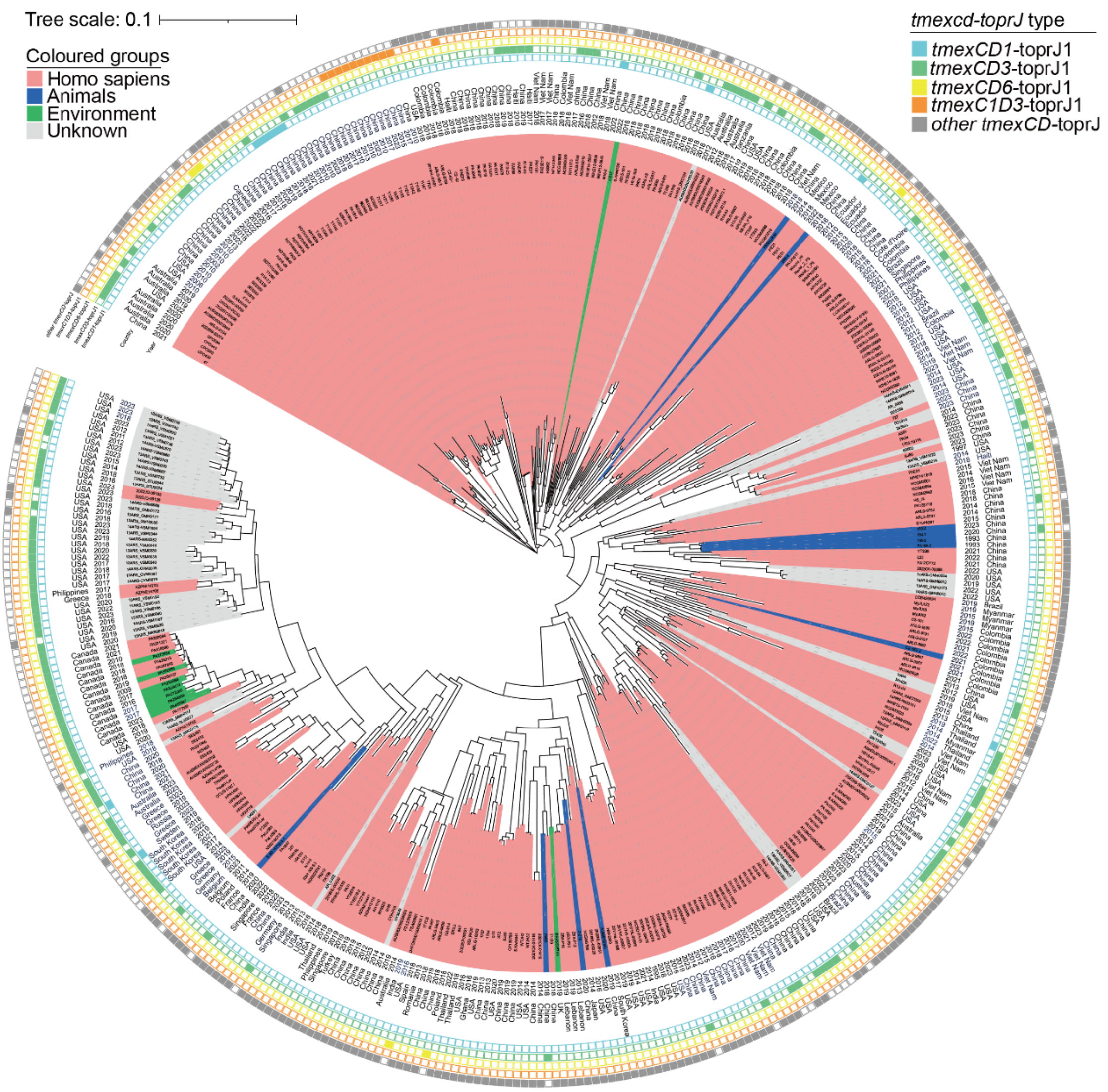

Figure S8. Phylogenetic analysis of *P. aeruginosa* carrying the *tmexCD-toprJ* gene cluster. Different background colors of strain names indicate different hosts. This is followed by year, country, and *tmexCD-toprJ* type.

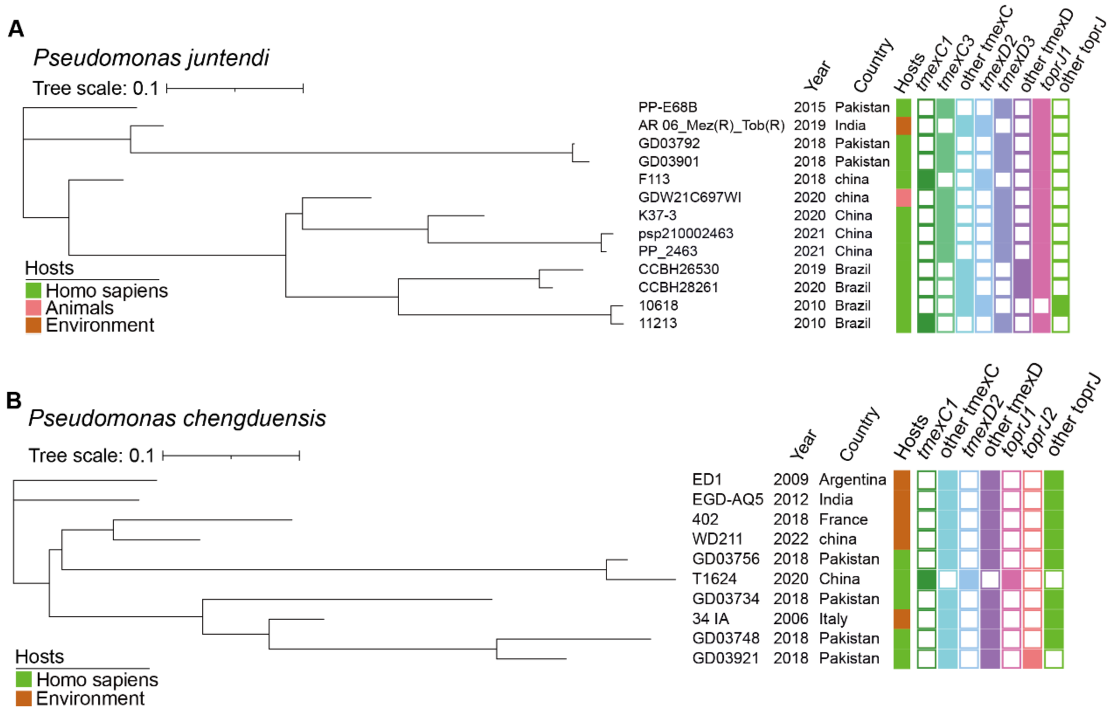

Figure S9. A phylogenetic tree representing the strains with the *tmexCD-toprJ* gene cluster. (A) Phylogenetic tree of 13 *P. juntendi* strains; (B) Phylogenetic tree of 10 *P. chengduensis* strains. The metadata of different isolates were arranged from left to right: year, country, hosts, and *tmexCD-toprJ* variant type.
