# Supplementary material for: Discovery and characterization of tmexCD3-toprJ1 on a plasmid from Pseudomonas putida isolated in a public trash can
Source: Microbiol Spectr. 2024 Aug 20;12(10):e00395-24. doi: 10.1128/spectrum.00395-24 (PMC11448381; doi:10.1128/spectrum.00395-24)
Supplement: Table S3 — Genomic information for all the pseudomonas strains. [file spectrum.00395-24-s0002.pdf]

Table S3. The genomic information of all the *pseudomonas* strains in this paper.

species	accession numbers	strain name	*tmexC*	*tmexD*	*toprJ*	years	source	country
Pseudomonas aeruginosa	JAVFFV000000000	47	*tmexC*	*tmexD*	*toprJ*	2021	Homo sapiens	China
Pseudomonas aeruginosa	JASUWJ000000000	CPO430	*tmexC3*	*tmexD3*	*toprJ1*	2020	Homo sapiens	Australia
Pseudomonas aeruginosa	JASUWB000000000	CPO383	*tmexC3*	*tmexD3*	*toprJ1*	2020	Homo sapiens	Australia
Pseudomonas aeruginosa	JASUWM000000000	CPO443	*tmexC3*	*tmexD3*	*toprJ1*	2020	Homo sapiens	Australia
Pseudomonas aeruginosa	JASUWC000000000	CPO384	*tmexC3*	*tmexD3*	*toprJ1*	2020	Homo sapiens	Australia
Pseudomonas aeruginosa	ABNXBP000000000	2022BB-00074	*tmexC3*	*tmexD3*	*toprJ*	2022	Homo sapiens	USA
Pseudomonas aeruginosa	DAHNSC000000000	ARLG-9134	*tmexC3*	*tmexD3*	*toprJ1*	2019	Homo sapiens	Australia
Pseudomonas aeruginosa	DAJRUK000000000	AUSMDU00025848	*tmexC*	*tmexD3*	*toprJ*	2019	Homo sapiens	Australia
Pseudomonas aeruginosa	DAFPGV000000000	AUSMDU00043779	*tmexC3*	*tmexD3*	*toprJ1*	2020	Homo sapiens	Australia
Pseudomonas aeruginosa	ABJEIM000000000	2022SY-00058	*tmexC3*	*tmexD2*	*toprJ1*	2022	Homo sapiens	USA
Pseudomonas aeruginosa	ABJQUF000000000	2022KU-00242	*tmexC*	*tmexD2*	*toprJ1*	2022	Homo sapiens	USA
Pseudomonas aeruginosa	ABJJFS000000000	2022KU-00255	*tmexC3*	*tmexD2*	*toprJ1*	2022	Homo sapiens	USA
Pseudomonas aeruginosa	JASTYF000000000	S.KANG48	*tmexC3*	*tmexD3*	*toprJ1*	2018	Homo sapiens	China
Pseudomonas aeruginosa	JADIPI000000000	XTI14	*tmexC*	*tmexD3*	*toprJ*	2020	Homo sapiens	China
Pseudomonas aeruginosa	JADISD000000000	SE5376	*tmexC*	*tmexD3*	*toprJ1*	2016	Homo sapiens	China
Pseudomonas aeruginosa	JADITA000000000	SE5349	*tmexC*	*tmexD3*	*toprJ1*	2017	Homo sapiens	China
Pseudomonas aeruginosa	JADITU000000000	SE5329	*tmexC*	*tmexD3*	*toprJ1*	2018	Homo sapiens	China
Pseudomonas aeruginosa	JADITL000000000	SE5338	*tmexC*	*tmexD3*	*toprJ1*	2017	Homo sapiens	China
Pseudomonas aeruginosa	JAUKGD000000000	R14-33	*tmexC3*	*tmexD3*	*toprJ1*	2023	Homo sapiens	China
Pseudomonas aeruginosa	JALKGR000000000	T1085	*tmexC3*	*tmexD3*	*toprJ1*	2013	Homo sapiens	China
Pseudomonas aeruginosa	CP064401-CP064402	NDTH10366	*tmexC6*	*tmexD6*	*toprJ1*	2020	Homo sapiens	China
Pseudomonas aeruginosa	CP136598-CP136599	PA12	*tmexC*	*tmexD*	*toprJ1*	2019	Homo sapiens	China
Pseudomonas aeruginosa	CP084321-CP084322	HS18-89	*tmexC*	*tmexD2*	*toprJ1*	2018	Homo sapiens	China
Pseudomonas aeruginosa	CP073080-CP073081	NDTH9845	*tmexC6*	*tmexD6*	*toprJ1*	2018	Homo sapiens	China
Pseudomonas aeruginosa	JAPFQJ000000000	PA23	*tmexC*	*tmexD2*	*toprJ1*	2021	Homo sapiens	Canada
Pseudomonas aeruginosa	JADHJP000000000	NDTH9845-2	*tmexC*	*tmexD2*	*toprJ1*	2018	Homo sapiens	China
Pseudomonas aeruginosa	JADHJN000000000	NDTH9434	*tmexC*	*tmexD2*	*toprJ1*	2017	Homo sapiens	China
Pseudomonas aeruginosa	JADHJQ000000000	NDTH9304	*tmexC*	*tmexD2*	*toprJ1*	2018	Homo sapiens	China
Pseudomonas aeruginosa	JADHJO000000000	NDTH10366-2	*tmexC*	*tmexD2*	*toprJ1*	2018	Homo sapiens	China
Pseudomonas aeruginosa	JAUKHV000000000	AR19639	*tmexC3*	*tmexD3*	*toprJ1*	2023	Homo sapiens	China
Pseudomonas aeruginosa	JALKGM000000000	T1251	*tmexC1*	*tmexD1*	*toprJ1*	2010	Homo sapiens	China
Pseudomonas aeruginosa	JALKGN000000000	T1248	*tmexC1*	*tmexD1*	*toprJ1*	2010	Homo sapiens	China
Pseudomonas aeruginosa	JALKGQ000000000	T1189	*tmexC1*	*tmexD1*	*toprJ1*	2010	Homo sapiens	China
Pseudomonas aeruginosa	JALKGK000000000	T1188	*tmexC1*	*tmexD1*	*toprJ1*	2010	Homo sapiens	China
Pseudomonas aeruginosa	JALKGP000000000	T1201	*tmexC1*	*tmexD1*	*toprJ1*	2010	Homo sapiens	China
Pseudomonas aeruginosa	JADIQW000000000	SE5410	*tmexC3*	*tmexD3*	*toprJ1*	2013	Homo sapiens	China
Pseudomonas aeruginosa	JAUKGS000000000	PA1804	*tmexC3*	*tmexD*	*toprJ1*	2023	Homo sapiens	China
Pseudomonas aeruginosa	JAUKGR000000000	PA1814	*tmexC3*	*tmexD3*	*toprJ1*	2023	Homo sapiens	China
Pseudomonas aeruginosa	JADIRT000000000	SE5387	*tmexC3*	*tmexD3*	*toprJ1*	2015	Homo sapiens	China
Pseudomonas aeruginosa	JADIPN000000000	SE5464	*tmexC3*	*tmexD3*	*toprJ1*	2010	Homo sapiens	China
Pseudomonas aeruginosa	JADITB000000000	SE5348	*tmexC3*	*tmexD3*	*toprJ1*	2017	Homo sapiens	China
Pseudomonas aeruginosa	JADISZ000000000	SE5350	*tmexC3*	*tmexD3*	*toprJ1*	2017	Homo sapiens	China
Pseudomonas aeruginosa	JALPNJ000000000	T1217	*tmexC1*	*tmexD3*	*toprJ1*	2010	Homo sapiens	China
Pseudomonas aeruginosa	JALPNL000000000	T1211	*tmexC1*	*tmexD3*	*toprJ1*	2010	Homo sapiens	China
Pseudomonas aeruginosa	JALPNH000000000	T1250	*tmexC1*	*tmexD3*	*toprJ1*	2010	Homo sapiens	China
Pseudomonas aeruginosa	JALKGL000000000	TA39	*tmexC1*	*tmexD1*	*toprJ1*	2002	Homo sapiens	China
Pseudomonas aeruginosa	JALPNO000000000	T1183	*tmexC1*	*tmexD3*	*toprJ1*	2010	Homo sapiens	China
Pseudomonas aeruginosa	JALPNK000000000	T1214	*tmexC1*	*tmexD3*	*toprJ1*	2010	Homo sapiens	China
Pseudomonas aeruginosa	JALPNI000000000	T1242	*tmexC1*	*tmexD3*	*toprJ1*	2010	Homo sapiens	China
Pseudomonas aeruginosa	JALPNN000000000	T1185	*tmexC1*	*tmexD3*	*toprJ1*	2010	Homo sapiens	China
Pseudomonas aeruginosa	JALPNM000000000	T1187	*tmexC1*	*tmexD3*	*toprJ1*	2010	Homo sapiens	China
Pseudomonas aeruginosa	JALPNG000000000	T725	*tmexC1*	*tmexD3*	*toprJ1*	2006	Homo sapiens	China
Pseudomonas aeruginosa	ABHYED000000000	2018HL-01561	*tmexC*	*tmexD2*	*toprJ*	2018	Homo sapiens	USA
Pseudomonas aeruginosa	DAHOOE000000000	ARLG-9813	*tmexC*	*tmexD2*	*toprJ*	2019	Homo sapiens	Colombia
Pseudomonas aeruginosa	DAHONS000000000	ARLG-9799	*tmexC*	*tmexD2*	*toprJ1*	2019	Homo sapiens	Colombia
Pseudomonas aeruginosa	SWGP00000000	C2-42-1	*tmexC*	*tmexD*	*toprJ1*	2014	Homo sapiens	Colombia

Pseudomonas aeruginosa	SWGQ00000000	C2-42	*tmexC*	*tmexD*	*toprJ1*	2014	Homo sapiens	Colombia
Pseudomonas aeruginosa	DAHPSF000000000	PAE23	*tmexC3*	*tmexD3*	*toprJ1*	2014	Homo sapiens	Haiti
Pseudomonas aeruginosa	JAUKHH000000000	PA1048	*tmexC*	*tmexD3*	*toprJ1*	2023	Homo sapiens	China
Pseudomonas aeruginosa	JAUKHG000000000	PA1049	*tmexC*	*tmexD3*	*toprJ1*	2023	Homo sapiens	China
Pseudomonas aeruginosa	JAUKHB000000000	PA1087	*tmexC*	*tmexD3*	*toprJ1*	2023	Homo sapiens	China
Pseudomonas aeruginosa	JAUKHL000000000	PA1019	*tmexC*	*tmexD3*	*toprJ1*	2023	Homo sapiens	China
Pseudomonas aeruginosa	JAUKHK000000000	PA1039	*tmexC*	*tmexD3*	*toprJ1*	2023	Homo sapiens	China
Pseudomonas aeruginosa	JAUKHN000000000	PA1017	*tmexC*	*tmexD3*	*toprJ1*	2023	Homo sapiens	China
Pseudomonas aeruginosa	JAUKHO000000000	PA975	*tmexC*	*tmexD3*	*toprJ1*	2023	Homo sapiens	China
Pseudomonas aeruginosa	JAUKGF000000000	R13-23	*tmexC3*	*tmexD3*	*toprJ1*	2023	Homo sapiens	China
Pseudomonas aeruginosa	JAUKHP000000000	PA868	*tmexC3*	*tmexD3*	*toprJ1*	2023	Homo sapiens	China
Pseudomonas aeruginosa	DAHPSD000000000	PAE40	*tmexC3*	*tmexD3*	*toprJ1*	2014	Homo sapiens	Haiti
Pseudomonas aeruginosa	JAUKGB000000000	R14-53	*tmexC3*	*tmexD3*	*toprJ1*	2023	Homo sapiens	China
Pseudomonas aeruginosa	DAHPSG000000000	PAE37	*tmexC3*	*tmexD3*	*toprJ1*	2014	Homo sapiens	Haiti
Pseudomonas aeruginosa	JAUKGW000000000	PA1191	*tmexC3*	*tmexD3*	*toprJ1*	2023	Homo sapiens	china
Pseudomonas aeruginosa	JARKMQ000000000	PA202110	*tmexC3*	*tmexD3*	*toprJ1*	2020	Homo sapiens	China
Pseudomonas aeruginosa	JAPCHR000000000	HS062	*tmexC3*	*tmexD3*	*toprJ1*	2018	Homo sapiens	China
Pseudomonas aeruginosa	CP114761-CP114762	NF143349	*tmexC*	*tmexD3*	*toprJ1*	2022	Homo sapiens	China
Pseudomonas aeruginosa	DAFQBB000000000	NCGM2998	*tmexC*	*tmexD2*	*toprJ*	2014	Homo sapiens	Viet Nam
Pseudomonas aeruginosa	DAFQAQ000000000	NCGM3009	*tmexC*	*tmexD2*	*toprJ*	2014	Homo sapiens	Viet Nam
Pseudomonas aeruginosa	CP096956-CP096957	NY11173	*tmexC*	*tmexD2*	*toprJ*	2019	Homo sapiens	China
Pseudomonas aeruginosa	DAHOXC000000000	ARLG-9789	*tmexC*	*tmexD2*	*toprJ1*	2019	Homo sapiens	Colombia
Pseudomonas aeruginosa	DAFQAS000000000	NCGM3010	*tmexC*	*tmexD2*	*toprJ*	2014	Homo sapiens	Viet Nam
Pseudomonas aeruginosa	BSBB00000000	NIHE14-2037	*tmexC*	*tmexD2*	*toprJ*	2014	Homo sapiens	Viet Nam
Pseudomonas aeruginosa	BSAR00000000	NIHE12-0889	*tmexC*	*tmexD2*	*toprJ*	2012	Homo sapiens	Viet Nam
Pseudomonas aeruginosa	BSAQ00000000	NIHE12-0840	*tmexC*	*tmexD2*	*toprJ*	2012	Homo sapiens	Viet Nam
Pseudomonas aeruginosa	JALKGV000000000	N16-2	*tmexC1*	*tmexD1*	*toprJ1*	2016	Homo sapiens	China
Pseudomonas aeruginosa	JAUAST000000000	C517	*tmexC*	*tmexD*	*toprJ1*	2023	environment	China
Pseudomonas aeruginosa	JASTXZ000000000	S.KANG59	*tmexC*	*tmexD3*	*toprJ1*	2019	Homo sapiens	China
Pseudomonas aeruginosa	JABFLW000000000	HS15-101	*tmexC*	*tmexD3*	*toprJ1*	2015	Homo sapiens	China
Pseudomonas aeruginosa	JAUZXS000000000	R0015	*tmexC*	*tmexD*	*toprJ1*	2021	Homo sapiens	China
Pseudomonas aeruginosa	JAMOGL000000000	NY5533	*tmexC*	*tmexD3*	*toprJ1*	2019	Homo sapiens	China
Pseudomonas aeruginosa	JABFLX000000000	HS15-106	*tmexC*	*tmexD*	*toprJ1*	2015	Homo sapiens	China
Pseudomonas aeruginosa	DAHPRP000000000	PA05	*tmexC*	*tmexD3*	*toprJ1*	2019	Homo sapiens	China
Pseudomonas aeruginosa	JABMEE000000000	KM18-18	*tmexC*	*tmexD3*	*toprJ1*	2018	Homo sapiens	China
Pseudomonas aeruginosa	DAHOZA000000000	ARLG-9917	*tmexC*	*tmexD2*	*toprJ1*	2019	Homo sapiens	Colombia
Pseudomonas aeruginosa	JAUKGQ000000000	PA2005	*tmexC3*	*tmexD3*	*toprJ1*	2023	Homo sapiens	China
Pseudomonas aeruginosa	DAHNVX000000000	ARLG-9401	*tmexC*	*tmexD2*	*toprJ*	2019	Homo sapiens	China
Pseudomonas aeruginosa	JALKGO000000000	T1246	*tmexC1*	*tmexD1*	*toprJ1*	2010	Homo sapiens	China
Pseudomonas aeruginosa	CP090649-CP090650	PA1609	*tmexC*	*tmexD3*	*toprJ1*	2015	Homo sapiens	China
Pseudomonas aeruginosa	DAFSHB000000000	14ARS_DMC124	*tmexC3*	*tmexD3*	*toprJ1*	2018	N/A	USA
Pseudomonas aeruginosa	DAJRUF000000000	AUSMDU00018333	*tmexC3*	*tmexD3*	*toprJ1*	2018	Homo sapiens	Australia
Pseudomonas aeruginosa	DAFPHN000000000	AUSMDU00018252	*tmexC3*	*tmexD3*	*toprJ1*	2018	Homo sapiens	Australia
Pseudomonas aeruginosa	DAJRUG000000000	AUSMDU00019048	*tmexC3*	*tmexD3*	*toprJ1*	2018	Homo sapiens	Australia
Pseudomonas aeruginosa	DAJRWG000000000	AUSMDU00019049	*tmexC3*	*tmexD3*	*toprJ1*	2018	Homo sapiens	Australia
Pseudomonas aeruginosa	ABOHSQ000000000	2023GN-00534	*tmexC*	*tmexD2*	*toprJ1*	2023	Homo sapiens	USA
Pseudomonas aeruginosa	ABMFVZ000000000	2023KU-00219	*tmexC3*	*tmexD3*	*toprJ1*	2023	Homo sapiens	USA
Pseudomonas aeruginosa	OVCV00000000	KCRI-321A	*tmexC3*	*tmexD*	*toprJ1*	2014	Homo sapiens	Tanzania
Pseudomonas aeruginosa	JAUKGC000000000	R14-43	*tmexC3*	*tmexD3*	*toprJ1*	2023	Homo sapiens	China
Pseudomonas aeruginosa	DAHOXU000000000	ARLG-9907	*tmexC*	*tmexD2*	*toprJ1*	2018	Homo sapiens	Colombia
Pseudomonas aeruginosa	JAUKFZ000000000	S13-35	*tmexC3*	*tmexD3*	*toprJ1*	2023	Homo sapiens	China
Pseudomonas aeruginosa	DAHOVM000000000	ARLG-9497	*tmexC3*	*tmexD3*	*toprJ1*	2019	Homo sapiens	China
Pseudomonas aeruginosa	CP086010-CP086015	KB-PA_F19	*tmexC*	*tmexD3*	*toprJ1*	2015	Homo sapiens	China
Pseudomonas aeruginosa	JADIPB000000000	YTSS7	*tmexC*	*tmexD3*	*toprJ1*	2020	Homo sapiens	China
Pseudomonas aeruginosa	JADIOZ000000000	YTSS9	*tmexC*	*tmexD3*	*toprJ1*	2020	Homo sapiens	China
Pseudomonas aeruginosa	DAFQBF000000000	NCGM2996	*tmexC*	*tmexD2*	*toprJ*	2014	Homo sapiens	China
Pseudomonas aeruginosa	DAFQAF000000000	NCGM3019	*tmexC*	*tmexD2*	*toprJ*	2014	Homo sapiens	Viet Nam
Pseudomonas aeruginosa	JAUKPN000000000	PA98zyfcau	*tmexC*	*tmexD*	*toprJ1*	2018	animals	China

Pseudomonas aeruginosa	JAGMUW000000000	PE21	*tmexC*	*tmexD2*	*toprJ1*	2013	Homo sapiens	Mexico
Pseudomonas aeruginosa	JARDUX000000000	PE83	*tmexC*	*tmexD2*	*toprJ1*	2015	Homo sapiens	Mexico
Pseudomonas aeruginosa	JARDUV000000000	PE21	*tmexC*	*tmexD2*	*toprJ1*	2013	Homo sapiens	Mexico
Pseudomonas aeruginosa	JACAKE000000000	294-4	*tmexC*	*tmexD3*	*toprJ1*	2019	Animals	China
Pseudomonas aeruginosa	CP016214-CP016215	PA121617	*tmexC1*	*tmexD1*	*toprJ1*	2012	Homo sapiens	China
Pseudomonas aeruginosa	JARNJS000000000	Wound_P8	*tmexC*	*tmexD2*	*toprJ1*	2022	Homo sapiens	Ecuador
Pseudomonas aeruginosa	JARNJU000000000	Rectal_2_P8	*tmexC*	*tmexD*	*toprJ*	2022	Homo sapiens	Ecuador
Pseudomonas aeruginosa	JARNJT000000000	Rectal_1_P8	*tmexC*	*tmexD*	*toprJ*	2022	Homo sapiens	Ecuador
Pseudomonas aeruginosa	CP132993-CP132994	SRMPA3860	*tmexC*	*tmexD2*	*toprJ1*	2021	Homo sapiens	China
Pseudomonas aeruginosa	CP095920-CP095921	AR19640	*tmexC6*	*tmexD6*	*toprJ1*	2021	Homo sapiens	China
Pseudomonas aeruginosa	JALLGZ000000000	AR19727	*tmexC*	*tmexD2*	*toprJ1*	2021	Homo sapiens	China
Pseudomonas aeruginosa	JALLHA000000000	AR19726	*tmexC*	*tmexD2*	*toprJ1*	2021	Homo sapiens	China
Pseudomonas aeruginosa	JALLGX000000000	AR23664	*tmexC*	*tmexD2*	*toprJ1*	2021	Homo sapiens	China
Pseudomonas aeruginosa	NRBZ00000000	Pa359	*tmexC3*	*tmexD3*	*toprJ1*	2015	Homo sapiens	Cote d'Ivoire
Pseudomonas aeruginosa	DAHOXB000000000	ARLG-9788	*tmexC*	*tmexD2*	*toprJ*	2019	Homo sapiens	Colombia
Pseudomonas aeruginosa	DAHONR000000000	ARLG-9794	*tmexC*	*tmexD2*	*toprJ1*	2019	Homo sapiens	Colombia
Pseudomonas aeruginosa	JAECUE000000000	CCBH26731	*tmexC*	*tmexD2*		2019	Homo sapiens	Brazil
Pseudomonas aeruginosa	DAFNXC000000000	15DU009025	*tmexC*	*tmexD3*	*toprJ1*	2015	Homo sapiens	Singapore
Pseudomonas aeruginosa	NSSR00000000	PhDW6	*tmexC*	*tmexD3*	*toprJ1*	1993	Homo sapiens	Philippines
Pseudomonas aeruginosa	LLUU00000000	WH-SGI-V-07300	*tmexC3*	*tmexD3*	*toprJ1*	1993	Homo sapiens	Philippines
Pseudomonas aeruginosa	ABEUTY000000000	2020CK-00187	*tmexC*	*tmexD3*	*toprJ1*	2020	Homo sapiens	USA
Pseudomonas aeruginosa	ABLFHV000000000	2023KU-00094	*tmexC1*	*tmexD2*	*toprJ1*	2023	Homo sapiens	USA
Pseudomonas aeruginosa	ABFHHY000000000	2021HL-01148	*tmexC*	*tmexD2*	*toprJ1*	2021	Homo sapiens	USA
Pseudomonas aeruginosa	ABMISH000000000	2022KU-00403	*tmexC*	*tmexD2*	*toprJ1*	2022	Homo sapiens	USA
Pseudomonas aeruginosa	ABJJRK000000000	2022SY-00069	*tmexC3*	*tmexD2*	*toprJ1*	2022	Homo sapiens	USA
Pseudomonas aeruginosa	JADPHX000000000	CCBH27928	*tmexC*	*tmexD2*	*toprJ*	2020	Homo sapiens	Brazil
Pseudomonas aeruginosa	DAHONV000000000	ARLG-9803	*tmexC*	*tmexD2*	*toprJ1*	2019	Homo sapiens	Colombia
Pseudomonas aeruginosa	ABGWYM00000000[illegible]	2022LN-00119	*tmexC3*	*tmexD2*	*toprJ1*	2022	Homo sapiens	USA
Pseudomonas aeruginosa	ABJFCH000000000	2022LN-00169	*tmexC3*	*tmexD2*	*toprJ1*	2022	Homo sapiens	USA
Pseudomonas aeruginosa	ABFXYP000000000	2021LN-00166	*tmexC3*	*tmexD2*	*toprJ1*	2021	Homo sapiens	USA
Pseudomonas aeruginosa	BSBD00000000	NIHE15-2081	*tmexC*	*tmexD2*	*toprJ*	2015	Homo sapiens	Viet Nam
Pseudomonas aeruginosa	BSBA00000000	NIHE14-1826	*tmexC*	*tmexD2*	*toprJ*	2014	Homo sapiens	Viet Nam
Pseudomonas aeruginosa	DAFQBL000000000	NCGM2990	*tmexC*	*tmexD2*	*toprJ*	2014	Homo sapiens	Viet Nam
Pseudomonas aeruginosa	DAFSFW000000000	14ARS-EVR0071	*tmexC3*	*tmexD3*	*toprJ1*	2018	N/A	USA
Pseudomonas aeruginosa	DAFSFU000000000	14ARS-GMH0014	*tmexC3*	*tmexD3*	*toprJ1*	2018	N/A	USA
Pseudomonas aeruginosa	CP027165-CP027170	AR_0356	*tmexC*	*tmexD*	*toprJ*	2018	N/A	USA
Pseudomonas aeruginosa	MT598646	SE5388-1	*tmexC3*	*tmexD3*	*toprJ1*	2015	N/A	China
Pseudomonas aeruginosa	MF344568	727	*tmexC3*	*tmexD3*	*toprJ1*	2014	Homo sapiens	China
Pseudomonas aeruginosa	MF344571	R31014	*tmexC3*	*tmexD3*	*toprJ1*	2015	Homo sapiens	China
Pseudomonas aeruginosa	MT598646	243931	*tmexC*	*tmexD3*	*toprJ1*	2016	N/A	China
Pseudomonas aeruginosa	MF344570	A681	*tmexC1*	*tmexD1*	*toprJ1*	2014	Homo sapiens	China
Pseudomonas aeruginosa	MH547560	PA34	*tmexC1*	*tmexD1*	*toprJ1*	1997	Homo sapiens	China
Pseudomonas aeruginosa	MN208061	1705-19119	*tmexC*	*tmexD2*	*toprJ*	2017	N/A	China
Pseudomonas aeruginosa	MN208063	60503	*tmexC*	*tmexD2*	*toprJ*	2016	N/A	China
Pseudomonas aeruginosa	CP133755-CP133756	BJ86	*tmexC*	*tmexD*	*toprJ*	2019	Homo sapiens	China
Pseudomonas aeruginosa	DAFSGS000000000	13ARS_VSM1025	*tmexC3*	*tmexD3*	*toprJ*	2018	N/A	USA
Pseudomonas aeruginosa	DAFSFA000000000	13ARS_VSM0214	*tmexC3*	*tmexD3*	*toprJ*	2018	N/A	USA
Pseudomonas aeruginosa	DAHPSH000000000	PAE01	*tmexC3*	*tmexD3*	*toprJ1*	2014	Homo sapiens	Haiti
Pseudomonas aeruginosa	BSAZ00000000	NIHE14-1818	*tmexC*	*tmexD2*	*toprJ*	2014	Homo sapiens	Viet Nam
Pseudomonas aeruginosa	DAFQAY000000000	NCGM3001	*tmexC*	*tmexD2*	*toprJ*	2014	Homo sapiens	Viet Nam
Pseudomonas aeruginosa	DAFQBI000000000	NCGM2994	*tmexC*	*tmexD2*	*toprJ*	2014	Homo sapiens	Viet Nam
Pseudomonas aeruginosa	DAFQBM000000000	NCGM2992	*tmexC*	*tmexD2*	*toprJ*	2014	Homo sapiens	Viet Nam
Pseudomonas aeruginosa	JAJUVP000000000	HS_74	*tmexC3*	*tmexD3*	*toprJ1*	2020	Homo sapiens	China
Pseudomonas aeruginosa	MKEM00000000	PA13SY16	*tmexC*	*tmexD*	*toprJ1*	2013	Homo sapiens	China
Pseudomonas aeruginosa	DAHOWI000000000	ARLG-9752	*tmexC*	*tmexD*	*toprJ1*	2019	Homo sapiens	China
Pseudomonas aeruginosa	DAHNXL000000000	ARLG-9737	*tmexC*	*tmexD*	*toprJ1*	2019	Homo sapiens	China
Pseudomonas aeruginosa	JASTXD000000000	S.KANG87	*tmexC*	*tmexD3*	*toprJ1*	2020	Homo sapiens	China
Pseudomonas aeruginosa	JACAKH000000000	159-2	*tmexC*	*tmexD3*	*toprJ1*	2019	Animals	China

Pseudomonas aeruginosa	JACAKG000000000	164-1	*tmexC*	*tmexD3*	*toprJ1*	2019	Animals	China
Pseudomonas aeruginosa	JACAKF000000000	166-2	*tmexC*	*tmexD3*	*toprJ1*	2019	Animals	China
Pseudomonas aeruginosa	JAKHEW000000000	PA166-2	*tmexC3*	*tmexD3*	*toprJ1*	2019	animals	China
Pseudomonas aeruginosa	JADIPA000000000	YTSS8	*tmexC*	*tmexD3*	*toprJ1*	2020	Homo sapiens	China
Pseudomonas aeruginosa	VWQL00000000	L23	*tmexC*	*tmexD3*	*toprJ1*	2013	Homo sapiens	China
Pseudomonas aeruginosa	MKEL00000000	PA12GY72	*tmexC*	*tmexD3*	*toprJ1*	2013	Homo sapiens	China
Pseudomonas aeruginosa	CP124658-CP124659	2022CK-00068	*tmexC*	*tmexD*	*toprJ*	2022	Homo sapiens	USA
Pseudomonas aeruginosa	DAFSET000000000	14ARS-GMH0054	*tmexC*	*tmexD3*	*toprJ*	2018	N/A	USA
Pseudomonas aeruginosa	DAFSFQ000000000	14ARS-GMH0012	*tmexC*	*tmexD3*	*toprJ1*	2018	N/A	USA
Pseudomonas aeruginosa	DAFSHU000000000	13ARS_GMH0170	*tmexC*	*tmexD3*	*toprJ1*	2018	N/A	USA
Pseudomonas aeruginosa	DAFSEU000000000	14ARS-GMH0013	*tmexC*	*tmexD3*	*toprJ*	2018	N/A	USA
Pseudomonas aeruginosa	JALLDX000000000	CCBH26694	*tmexC*	*tmexD*	*toprJ*	2019	Homo sapiens	Brazil
Pseudomonas aeruginosa	DAFQDU000000000	MyJU122	*tmexC*	*tmexD*	*toprJ1*	2016	Homo sapiens	Myanmar
Pseudomonas aeruginosa	DAFQDX000000000	MyJU65	*tmexC*	*tmexD*	*toprJ1*	2016	Homo sapiens	Myanmar
Pseudomonas aeruginosa	DAFQDY000000000	MyJU62	*tmexC*	*tmexD*	*toprJ1*	2016	Homo sapiens	Myanmar
Pseudomonas aeruginosa	SWGT00000000	C2-101	*tmexC*	*tmexD*	*toprJ1*	2014	Homo sapiens	Colombia
Pseudomonas aeruginosa	DAHOXP000000000	ARLG-9895	*tmexC*	*tmexD2*	*toprJ1*	2019	Homo sapiens	Colombia
Pseudomonas aeruginosa	DAHOXD000000000	ARLG-9791	*tmexC*	*tmexD2*	*toprJ1*	2019	Homo sapiens	Colombia
Pseudomonas aeruginosa	DAHONP000000000	ARLG-9797	*tmexC*	*tmexD2*	*toprJ*	2019	Homo sapiens	Colombia
Pseudomonas aeruginosa	DAHOXS000000000	ARLG-9897	*tmexC*	*tmexD2*	*toprJ1*	2019	Homo sapiens	Colombia
Pseudomonas aeruginosa	SWGG00000000	C2-155-2	*tmexC*	*tmexD*	*toprJ1*	2015	animals	Colombia
Pseudomonas aeruginosa	DAHONZ000000000	ARLG-9807	*tmexC*	*tmexD2*	*toprJ1*	2019	Homo sapiens	Colombia
Pseudomonas aeruginosa	DAHPAV000000000	ARLG-9857	*tmexC*	*tmexD2*	*toprJ1*	2019	Homo sapiens	Colombia
Pseudomonas aeruginosa	DAHOOF000000000	ARLG-9814	*tmexC*	*tmexD2*	*toprJ1*	2019	Homo sapiens	Colombia
Pseudomonas aeruginosa	DAFPZZ000000000	NCGM3028	*tmexC*	*tmexD2*	*toprJ*	2014	Homo sapiens	Viet Nam
Pseudomonas aeruginosa	JAOVCX000000000	VA54	*tmexC*	*tmexD2*	*toprJ1*	2014	Homo sapiens	USA
Pseudomonas aeruginosa	MKEO00000000	M140A	*tmexC*	*tmexD*	*toprJ1*	2012	Homo sapiens	China
Pseudomonas aeruginosa	JAUKGG000000000	R12-04	*tmexC3*	*tmexD3*	*toprJ1*	2023	Homo sapiens	China
Pseudomonas aeruginosa	DAFSGX000000000	13ARS_RMC0008	*tmexC3*	*tmexD3*	*toprJ1*	2018	N/A	USA
Pseudomonas aeruginosa	DAFSFK000000000	14ARS-NKI0191	*tmexC*	*tmexD3*	*toprJ*	2018	N/A	USA
Pseudomonas aeruginosa	BSAS00000000	NIHE13-1183	*tmexC*	*tmexD2*	*toprJ*	2013	Homo sapiens	Viet Nam
Pseudomonas aeruginosa	DAFQAG000000000	NCGM3023	*tmexC*	*tmexD2*	*toprJ*	2014	Homo sapiens	Viet Nam
Pseudomonas aeruginosa	DAFSHL000000000	13ARS_MMH0094	*tmexC3*	*tmexD3*	*toprJ1*	2018	N/A	USA
Pseudomonas aeruginosa	DAFSHI000000000	13ARS_MMH0119	*tmexC3*	*tmexD3*	*toprJ1*	2018	N/A	USA
Pseudomonas aeruginosa	DAFQCZ000000000	MyJU5	*tmexC3*	*tmexD3*	*toprJ1*	2015	Homo sapiens	Myanmar
Pseudomonas aeruginosa	NSPM00000000	H638	*tmexC3*	*tmexD3*	*toprJ1*	2013	Homo sapiens	Thailand
Pseudomonas aeruginosa	CP039988-CP039989	T2436	*tmexC1*	*tmexD1*	*toprJ1*	2013	Homo sapiens	Thailand
Pseudomonas aeruginosa	NSPL00000000	B9(T2436)	*tmexC3*	*tmexD3*	*toprJ1*	2013	Homo sapiens	Thailand
Pseudomonas aeruginosa	JAUKGU000000000	PA1258	*tmexC3*	*tmexD3*	*toprJ1*	2023	Homo sapiens	China
Pseudomonas aeruginosa	ABNGIJ000000000	NA	*tmexC*	*tmexD2*	*toprJ1*	2023	Homo sapiens	USA
Pseudomonas aeruginosa	JABFOK000000000	HS18-41	*tmexC*	*tmexD3*	*toprJ1*	2018	Homo sapiens	China
Pseudomonas aeruginosa	ABMKJS000000000	2023HL-00648	*tmexC3*	*tmexD3*	*toprJ1*	2023	Homo sapiens	USA
Pseudomonas aeruginosa	BSAM00000000	NIHE11-0517	*tmexC*	*tmexD2*	*toprJ*	2011	Homo sapiens	Viet Nam
Pseudomonas aeruginosa	DAFQAU000000000	NCGM3004	*tmexC3*	*tmexD3*	*toprJ1*	2014	Homo sapiens	Viet Nam
Pseudomonas aeruginosa	DAFSFX000000000	14ARS-DMC0127	*tmexC3*	*tmexD3*	*toprJ1*	2018	N/A	USA
Pseudomonas aeruginosa	ABNXCN000000000	2022BB-00066	*tmexC3*	*tmexD*	*toprJ1*	2022	Homo sapiens	USA
Pseudomonas aeruginosa	DAFPEV000000000	AUSMDU00039924	*tmexC3*	*tmexD3*	*toprJ1*	2019	Homo sapiens	Australia
Pseudomonas aeruginosa	JAVFGU000000000	23	*tmexC3*	*tmexD3*	*toprJ1*	2021	Homo sapiens	China
Pseudomonas aeruginosa	JASTYL000000000	S.KANG40	*tmexC3*	*tmexD3*	*toprJ1*	2017	Homo sapiens	China
Pseudomonas aeruginosa	JASTXX000000000	S.KANG60	*tmexC3*	*tmexD3*	*toprJ1*	2019	Homo sapiens	China
Pseudomonas aeruginosa	JASTXU000000000	S.KANG65	*tmexC3*	*tmexD3*	*toprJ1*	2019	Homo sapiens	China
Pseudomonas aeruginosa	CP129995-CP129997	PA1045	*tmexC*	*tmexD*	*toprJ*	2023	Homo sapiens	China
Pseudomonas aeruginosa	JAUKHD000000000	PA1058	*tmexC*	*tmexD*	*toprJ*	2023	Homo sapiens	China
Pseudomonas aeruginosa	JAUKGZ000000000	PA1102	*tmexC*	*tmexD*	*toprJ*	2023	Homo sapiens	China
Pseudomonas aeruginosa	JAUKGO000000000	PA2034	*tmexC*	*tmexD*	*toprJ*	2023	Homo sapiens	China
Pseudomonas aeruginosa	JAUKHJ000000000	PA1040	*tmexC*	*tmexD*	*toprJ*	2023	Homo sapiens	China
Pseudomonas aeruginosa	JAUKHI000000000	PA1042	*tmexC*	*tmexD*	*toprJ*	2023	Homo sapiens	China
Pseudomonas aeruginosa	DAFPCG000000000	AUSMDU00025847	*tmexC*	*tmexD2*	*toprJ*	2019	Homo sapiens	Australia

Pseudomonas aeruginosa	JADIRS000000000	SE5388	*tmexC*	*tmexD3*	*toprJ1*	2015	Homo sapiens	China
Pseudomonas aeruginosa	JAJJII000000000	HS110	*tmexC*	*tmexD3*	*toprJ1*	2021	Homo sapiens	China
Pseudomonas aeruginosa	JAJJIF000000000	HS90	*tmexC*	*tmexD3*	*toprJ1*	2021	Homo sapiens	China
Pseudomonas aeruginosa	JAMKDJ000000000	CCBH29264	*tmexC*	*tmexD2*	*toprJ*	2020	Homo sapiens	Brazil
Pseudomonas aeruginosa	JALLCK000000000	CCBH29235	*tmexC*	*tmexD2*	*toprJ*	2020	Homo sapiens	Brazil
Pseudomonas aeruginosa	DAFSGB000000000	14ARS-BRH0051	*tmexC3*	*tmexD3*	*toprJ1*	2018	N/A	USA
Pseudomonas aeruginosa	DAFSEY000000000	13ARS_VSM0627	*tmexC*	*tmexD*	*toprJ*	2018	N/A	USA
Pseudomonas aeruginosa	DAFSDY000000000	14ARS-VSM1050	*tmexC*	*tmexD3*	*toprJ*	2018	N/A	USA
Pseudomonas aeruginosa	DARMAV000000000	PA-3	*tmexC*	*tmexD2*	*toprJ*	2016	Homo sapiens	China
Pseudomonas aeruginosa	DAHOVJ000000000	ARLG-9491	*tmexC*	*tmexD2*	*toprJ*	2019	Homo sapiens	China
Pseudomonas aeruginosa	JAUKGE000000000	R13-94	*tmexC3*	*tmexD3*	*toprJ1*	2023	Homo sapiens	China
Pseudomonas aeruginosa	JAUKGA000000000	R16-49	*tmexC3*	*tmexD3*	*toprJ1*	2023	Homo sapiens	China
Pseudomonas aeruginosa	JAUKHC000000000	PA1072	*tmexC3*	*tmexD3*	*toprJ1*	2023	Homo sapiens	China
Pseudomonas aeruginosa	JAUKHM000000000	PA1018	*tmexC3*	*tmexD3*	*toprJ1*	2023	Homo sapiens	China
Pseudomonas aeruginosa	JAUKGV000000000	PA1220	*tmexC3*	*tmexD3*	*toprJ1*	2023	Homo sapiens	China
Pseudomonas aeruginosa	JAUKGX000000000	PA1120	*tmexC3*	*tmexD3*	*toprJ1*	2023	Homo sapiens	China
Pseudomonas aeruginosa	BSAL00000000	NIHE11-0516	*tmexC*	*tmexD2*	*toprJ*	2011	Homo sapiens	Viet Nam
Pseudomonas aeruginosa	BSAP00000000	NIHE12-0719	*tmexC*	*tmexD2*	*toprJ*	2012	Homo sapiens	Viet Nam
Pseudomonas aeruginosa	BSAN00000000	NIHE12-0641	*tmexC*	*tmexD2*	*toprJ*	2012	Homo sapiens	Viet Nam
Pseudomonas aeruginosa	JADIUJ000000000	CZ1040	*tmexC3*	*tmexD3*	*toprJ1*	2018	Homo sapiens	China
Pseudomonas aeruginosa	JAUKGY000000000	PA1107	*tmexC*	*tmexD*	*toprJ*	2023	Homo sapiens	China
Pseudomonas aeruginosa	JAUKHA000000000	PA1090	*tmexC*	*tmexD*	*toprJ2*	2023	Homo sapiens	China
Pseudomonas aeruginosa	JAUKHE000000000	PA1052	*tmexC*	*tmexD*	*toprJ*	2023	Homo sapiens	China
Pseudomonas aeruginosa	JAUKHF000000000	PA1050	*tmexC*	*tmexD*	*toprJ*	2023	Homo sapiens	China
Pseudomonas aeruginosa	JADIRV000000000	SE5385	*tmexC*	*tmexD3*	*toprJ1*	2015	Homo sapiens	China
Pseudomonas aeruginosa	DAFQAI000000000	NCGM3017	*tmexC*	*tmexD2*	*toprJ*	2014	Homo sapiens	Viet Nam
Pseudomonas aeruginosa	JABMEF000000000	GZ18-2	*tmexC*	*tmexD*	*toprJ1*	2018	Homo sapiens	China
Pseudomonas aeruginosa	JADIVR000000000	12731	*tmexC3*	*tmexD3*	*toprJ1*	2016	Homo sapiens	China
Pseudomonas aeruginosa	CP089238-CP089239	JNQH-PA033	*tmexC*	*tmexD2*	*toprJ1*	2019	Homo sapiens	China
Pseudomonas aeruginosa	ABEURG000000000	2020HL-00733	*tmexC*	*tmexD*	*toprJ*	2020	Homo sapiens	USA
Pseudomonas aeruginosa	ABKQLC000000000	2023HL-00079	*tmexC1*	*tmexD2*	*toprJ*	2020	Homo sapiens	USA
Pseudomonas aeruginosa	ABKQLE000000000	2023HL-00078	*tmexC1*	*tmexD2*	*toprJ*	2022	Homo sapiens	USA
Pseudomonas aeruginosa	ABNUJO000000000	2023HL-00475	*tmexC*	*tmexD2*	*toprJ*	2023	Homo sapiens	USA
Pseudomonas aeruginosa	JAACJB000000000	NICED-PA-01	*tmexC*	*tmexD2*	*toprJ*	2016	Homo sapiens	India
Pseudomonas aeruginosa	ABEUSX000000000	2017HL-00506	*tmexC*	*tmexD2*	*toprJ*	2017	Homo sapiens	USA
Pseudomonas aeruginosa	ABEUSV000000000	2017HL-00505	*tmexC*	*tmexD2*	*toprJ*	2018	Homo sapiens	USA
Pseudomonas aeruginosa	ABEUSY000000000	2017HL-00507	*tmexC*	*tmexD2*	*toprJ*	2017	Homo sapiens	USA
Pseudomonas aeruginosa	ABEUTB000000000	2017HL-00508	*tmexC*	*tmexD2*	*toprJ*	2017	Homo sapiens	USA
Pseudomonas aeruginosa	CP137486	HPA2660	*tmexC*	*tmexD*	*toprJ*	2022	Homo sapiens	South Korea
Pseudomonas aeruginosa	JAGSOC000000000	AHM8C91AI	*tmexC3*	*tmexD3*	*toprJ*	2018	animals	China
Pseudomonas aeruginosa	CP071947-CP071949	2020HL-00861	*tmexC*	*tmexD3*	*toprJ*	2020	Homo sapiens	USA
Pseudomonas aeruginosa	ABEUSU000000000	2018HL-00229	*tmexC3*	*tmexD3*	*toprJ*	2018	Homo sapiens	USA
Pseudomonas aeruginosa	DAHPSW000000000	NCGM3798	*tmexC*	*tmexD*	*toprJ*	2017	Homo sapiens	Japan
Pseudomonas aeruginosa	JASAOR000000000	2-216	*tmexC*	*tmexD*	*toprJ*	2020	animals	China
Pseudomonas aeruginosa	CP061779	ZBX-P12	*tmexC*	*tmexD*	*toprJ*	2018	Homo sapiens	Lebanon
Pseudomonas aeruginosa	CP061777	ZBX-P23	*tmexC*	*tmexD*	*toprJ*	2018	Homo sapiens	Lebanon
Pseudomonas aeruginosa	JACWGV000000000	ZBX-P16	*tmexC*	*tmexD*	*toprJ*	2018	Homo sapiens	Lebanon
Pseudomonas aeruginosa	CAUPKU000000000	4_bin.2_MetaWRAP_	*tmexC*	*tmexD2*	*toprJ*	2023	environmental	UK
Pseudomonas aeruginosa	JALKHP000000000	T1150	*tmexC1*	*tmexD1*	*toprJ*	2009	Homo sapiens	China
Pseudomonas aeruginosa	CP024630-CP024631	PA59	*tmexC3*	*tmexD*	*toprJ*	2010	animals	China
Pseudomonas aeruginosa	ABEUHD000000000	2021CK-01416	*tmexC*	*tmexD*	*toprJ*	2021	Homo sapiens	USA
Pseudomonas aeruginosa	ABEUEE000000000	2021CK-01419	*tmexC*	*tmexD*	*toprJ*	2021	Homo sapiens	USA
Pseudomonas aeruginosa	CP104588-CP104589	WTJH32	*tmexC*	*tmexD3*	*toprJ*	2018	Homo sapiens	China
Pseudomonas aeruginosa	CP104584-CP104585	WTJH2	*tmexC*	*tmexD3*	*toprJ*	2018	Homo sapiens	China
Pseudomonas aeruginosa	JASXTF000000000	S.KANG41	*tmexC*	*tmexD3*	*toprJ*	2018	Homo sapiens	China
Pseudomonas aeruginosa	CP133755-CP133756	BJ86	*tmexC*	*tmexD*	*toprJ*	2019	Homo sapiens	China
Pseudomonas aeruginosa	JAFBLL000000000	S72	*tmexC3*	*tmexD3*	*toprJ*	2017	Homo sapiens	China
Pseudomonas aeruginosa	DAIHCI000000000	S75	*tmexC3*	*tmexD3*	*toprJ*	2016	Homo sapiens	USA

Pseudomonas aeruginosa	JALKGT000000000	1704	*tmexC1*	*tmexD1*	*toprJ*	2017	Homo sapiens	China
Pseudomonas aeruginosa	JALKGS000000000	1707	*tmexC1*	*tmexD1*	*toprJ*	2017	Homo sapiens	China
Pseudomonas aeruginosa	DAHPCS000000000	ARLG-10203	*tmexC*	*tmexD2*	*toprJ*	2019	Homo sapiens	USA
Pseudomonas aeruginosa	JAWLOI000000000	SSI_PA30	*tmexC3*	*tmexD3*	*toprJ*	2020	Homo sapiens	Ghana
Pseudomonas aeruginosa	ABKGII000000000	2022EP-00021	*tmexC1*	*tmexD2*	*toprJ*	2022	Homo sapiens	USA
Pseudomonas aeruginosa	JAUZBJ000000000	547	*tmexC*	*tmexD*	*toprJ*	2022	Homo sapiens	Thailand
Pseudomonas aeruginosa	JAHWUZ0000000000	ER5.2	*tmexC*	*tmexD*	*toprJ*	2020	Homo sapiens	Thailand
Pseudomonas aeruginosa	CP035739	1334/14	*tmexC6*	*tmexD6*	*toprJ*	2014	Homo sapiens	Poland
Pseudomonas aeruginosa	DAHNWB0000000000	ARLG-9406	*tmexC3*	*tmexD3*	*toprJ*	2019	Homo sapiens	China
Pseudomonas aeruginosa	DAHNVY000000000	ARLG-9403	*tmexC3*	*tmexD3*	*toprJ*	2019	Homo sapiens	China
Pseudomonas aeruginosa	JAUKHQ000000000	PA667	*tmexC*	*tmexD3*	*toprJ*	2023	Homo sapiens	China
Pseudomonas aeruginosa	JAMKXO000000000	177CS	*tmexC3*	*tmexD3*	*toprJ*	2019	Homo sapiens	Romania
Pseudomonas aeruginosa	DAFQKW000000000	NA	*tmexC6*	*tmexD6*	*toprJ*	2016	Homo sapiens	Spain
Pseudomonas aeruginosa	DARSIO000000000	PSA02961	*tmexC*	*tmexD*	*toprJ*	2023	Homo sapiens	USA
Pseudomonas aeruginosa	JMBR00000000	P2-L230/95	*tmexC3*	*tmexD3*	*toprJ*	1995	Homo sapiens	India
Pseudomonas aeruginosa	DAFPJZ000000000	AUSMDU00024056	*tmexC3*	*tmexD3*	*toprJ*	2018	Homo sapiens	Australia
Pseudomonas aeruginosa	CP124612	NY3649	*tmexC*	*tmexD*	*toprJ*	2014	Homo sapiens	China
Pseudomonas aeruginosa	DAFOIW000000000	CYH-P05	*tmexC*	*tmexD*	*toprJ*	2021	NA	China
Pseudomonas aeruginosa	JADIVY000000000	9148	*tmexC*	*tmexD*	*toprJ*	2014	Homo sapiens	China
Pseudomonas aeruginosa	CP096945	NY5525	*tmexC*	*tmexD*	*toprJ*	2019	Homo sapiens	China
Pseudomonas aeruginosa	JADIUW000000000	4011110	*tmexC*	*tmexD*	*toprJ*	2019	Homo sapiens	China
Pseudomonas aeruginosa	DAHMMU0000000000	IHMA_1344658	*tmexC*	*tmexD3*	*toprJ*	2015	Homo sapiens	Turkey
Pseudomonas aeruginosa	DAFOAD000000000	14DM013115	*tmexC3*	*tmexD3*	*toprJ*	2014	Homo sapiens	Singapore
Pseudomonas aeruginosa	JTYI00000000	AZPAE14441	*tmexC3*	*tmexD*	*toprJ*	2012	Homo sapiens	Philippines
Pseudomonas aeruginosa	CP045552	YT12746	*tmexC1*	*tmexD1*	*toprJ*	2012	Homo sapiens	China
Pseudomonas aeruginosa	JADIPC000000000	YTSEY8-2	*tmexC3*	*tmexD3*	*toprJ*	2020	Homo sapiens	China
Pseudomonas aeruginosa	CP054581	YTSEY8	*tmexC*	*tmexD3*	*toprJ*	2021	Homo sapiens	China
Pseudomonas aeruginosa	SAMN30433218	PA11	*tmexC3*	*tmexD3*	*toprJ*	2017	Homo sapiens	Thailand
Pseudomonas aeruginosa	ABHYFR000000000	2018HL-01538	*tmexC3*	*tmexD*	*toprJ*	2018	Homo sapiens	USA
Pseudomonas aeruginosa	ABEUSQ000000000	2018HL-00530	*tmexC3*	*tmexD*	*toprJ2*	2018	Homo sapiens	USA
Pseudomonas aeruginosa	CP027172-CP027173	AR_0353	*tmexC*	*tmexD*	*toprJ*	2018	N/A	USA
Pseudomonas aeruginosa	WOAL00000000	PA198	*tmexC*	*tmexD2*	*toprJ1*	2017	Homo sapiens	India
Pseudomonas aeruginosa	WOAF00000000	PA219	*tmexC*	*tmexD2*	*toprJ1*	2018	Homo sapiens	India
Pseudomonas aeruginosa	DAFOBJ000000000	14DB023741	*tmexC3*	*tmexD2*	*toprJ1*	2014	Homo sapiens	Singapore
Pseudomonas aeruginosa	CARUXP000000000	NA	*tmexC*	*tmexD*	*toprJ*	2022	Homo sapiens	Germany
Pseudomonas aeruginosa	JALKHC000000000	N18-5	*tmexC1*	*tmexD2*	*toprJ1*	2018	Homo sapiens	China
Pseudomonas aeruginosa	DAIHCG000000000	S117	*tmexC*	*tmexD2*	*toprJ1*	2018	Homo sapiens	China
Pseudomonas aeruginosa	VTFF00000000	164130	*tmexC*	*tmexD2*	*toprJ1*	2016	Homo sapiens	France
Pseudomonas aeruginosa	DAFODX000000000	PA0190	*tmexC3*	*tmexD2*	*toprJ1*	2012	Homo sapiens	Singapore
Pseudomonas aeruginosa	JAFCHE000000000	227	*tmexC*	*tmexD*	*toprJ*	2015	Homo sapiens	India
Pseudomonas aeruginosa	JAPZKC000000000	PA1847	*tmexC3*	*tmexD3*	*toprJ1*	2010	Homo sapiens	China
Pseudomonas aeruginosa	JAOXKX000000000	E-20-15715-4-1	*tmexC*	*tmexD*	*toprJ1*	2020	animals	France
Pseudomonas aeruginosa	JAFFVV000000000	NMI2145/13	*tmexC3*	*tmexD3*	*toprJ*	2013	Homo sapiens	Poland
Pseudomonas aeruginosa	CP075750	PaLo563	*tmexC*	*tmexD3*	*toprJ*	2018	Homo sapiens	Belgium
Pseudomonas aeruginosa	CP075748	PaLo565	*tmexC*	*tmexD3*	*toprJ1*	2018	Homo sapiens	Belgium
Pseudomonas aeruginosa	DAIWUL000000000	F2259	*tmexC3*	*tmexD3*	*toprJ1*	2015	Homo sapiens	Germany
Pseudomonas aeruginosa	JAKMHE000000000	Pae8709-Lar	*tmexC3*	*tmexD*	*toprJ1*	2018	Homo sapiens	Greece
Pseudomonas aeruginosa	JAKMHD000000000	Pae9938-Lar	*tmexC3*	*tmexD*	*toprJ1*	2018	Homo sapiens	Greece
Pseudomonas aeruginosa	RXFP00000000	U4581	*tmexC*	*tmexD3*	*toprJ1*	2018	N/A	USA
Pseudomonas aeruginosa	CP137522-CP137523	HPA0118	*tmexC3*	*tmexD3*	*toprJ1*	2021	Homo sapiens	South Korea
Pseudomonas aeruginosa	CP137500-CP137503	HPA0384	*tmexC3*	*tmexD3*	*toprJ1*	2021	Homo sapiens	South Korea
Pseudomonas aeruginosa	CP137505-CP137506	HPA0044	*tmexC3*	*tmexD3*	*toprJ1*	2021	Homo sapiens	South Korea
Pseudomonas aeruginosa	CP137495-CP137497	HPA0875	*tmexC3*	*tmexD3*	*toprJ1*	2021	Homo sapiens	South Korea
Pseudomonas aeruginosa	CP137491-CP137492	HPA1406	*tmexC3*	*tmexD3*	*toprJ1*	2022	Homo sapiens	South Korea
Pseudomonas aeruginosa	CP043328	CCUG 51971	*tmexC1*	*tmexD1*	*toprJ1*	2001	Homo sapiens	Sweden
Pseudomonas aeruginosa	JAKMHF000000000	Pae90-Lar	*tmexC3*	*tmexD3*	*toprJ1*	2018	Homo sapiens	Greece
Pseudomonas aeruginosa	LYTU00000000	TRN6609	*tmexC3*	*tmexD*	*toprJ*	2012	Homo sapiens	Russia
Pseudomonas aeruginosa	JTWX00000000	AZPAE14708	*tmexC3*	*tmexD3*	*toprJ1*	2012	Homo sapiens	Greece

Pseudomonas aeruginosa	JTWZ00000000	AZPAE14706	*tmexC3*	*tmexD3*	*toprJ1*	2012	Homo sapiens	Greece
Pseudomonas aeruginosa	DAJRUI000000000	AUSMDU00022728	*tmexC3*	*tmexD3*	*toprJ1*	2018	Homo sapiens	Australia
Pseudomonas aeruginosa	DAFPJU000000000	AUSMDU00022702	*tmexC3*	*tmexD3*	*toprJ1*	2018	Homo sapiens	Australia
Pseudomonas aeruginosa	CP054845	SE5429	*tmexC3*	*tmexD3*	*toprJ*	2012	Homo sapiens	China
Pseudomonas aeruginosa	CP046406	SE5458	*tmexC1*	*tmexD1*	*toprJ1*	2011	Homo sapiens	China
Pseudomonas aeruginosa	JARKMF000000000	PA201945	*tmexC3*	*tmexD3*	*toprJ1*	2019	Homo sapiens	China
Pseudomonas aeruginosa	JADIQM000000000	SE5432	*tmexC3*	*tmexD3*	*toprJ1*	2012	Homo sapiens	China
Pseudomonas aeruginosa	JADIPL000000000	SE5467	*tmexC3*	*tmexD3*	*toprJ1*	2010	Homo sapiens	China
Pseudomonas aeruginosa	DAFSGZ000000000	13ARS_NMC0175	*tmexC3*	*tmexD3*	*toprJ1*	2018	N/A	USA
Pseudomonas aeruginosa	JTXD00000000	AZPAE14702	*tmexC*	*tmexD*	*toprJ1*	2012	Homo sapiens	Philippines
Pseudomonas aeruginosa	DAFSFI000000000	14ARS-SLH0037	*tmexC3*	*tmexD3*	*toprJ1*	2018	NA	USA
Pseudomonas aeruginosa	DAFSHJ000000000	13ARS_MMH0117	*tmexC3*	*tmexD3*	*toprJ1*	2018	N/A	USA
Pseudomonas aeruginosa	DAFSDA000000000	PA177356	*tmexC3*	*tmexD3*	*toprJ1*	2016	Homo sapiens	Canada
Pseudomonas aeruginosa	DAFSCS000000000	PA405987	*tmexC3*	*tmexD3*	*toprJ1*	2017	environment	Canada
Pseudomonas aeruginosa	DAFSCN000000000	PA250989	*tmexC3*	*tmexD3*	*toprJ1*	2018	environment	Canada
Pseudomonas aeruginosa	DAFSCG000000000	PA272080	*tmexC3*	*tmexD3*	*toprJ1*	2018	environment	Canada
Pseudomonas aeruginosa	DAFSCO000000000	PA539413	*tmexC3*	*tmexD3*	*toprJ1*	2018	environment	Canada
Pseudomonas aeruginosa	DAFSCT000000000	PA250988	*tmexC3*	*tmexD3*	*toprJ1*	2018	environment	Canada
Pseudomonas aeruginosa	DAFSCU000000000	PA350137	*tmexC3*	*tmexD3*	*toprJ1*	2017	Homo sapiens	Canada
Pseudomonas aeruginosa	DAFSCZ000000000	PA392895	*tmexC3*	*tmexD3*	*toprJ1*	2017	environment	Canada
Pseudomonas aeruginosa	DAFSCR000000000	PA287892	*tmexC3*	*tmexD3*	*toprJ1*	2017	Homo sapiens	Canada
Pseudomonas aeruginosa	DAFSCD000000000	PA426218	*tmexC3*	*tmexD3*	*toprJ1*	2019	Homo sapiens	Canada
Pseudomonas aeruginosa	DAFSCJ000000000	PA273658	*tmexC3*	*tmexD3*	*toprJ1*	2018	environment	Canada
Pseudomonas aeruginosa	DAFSBZ000000000	PA418590	*tmexC3*	*tmexD3*	*toprJ1*	2019	Homo sapiens	Canada
Pseudomonas aeruginosa	DAFSCE000000000	PA371271	*tmexC3*	*tmexD3*	*toprJ1*	2019	Homo sapiens	Canada
Pseudomonas aeruginosa	DAFSCV000000000	PA350884	*tmexC3*	*tmexD3*	*toprJ1*	2017	Homo sapiens	Canada
Pseudomonas aeruginosa	DAFSHO000000000	13ARS_MAR0018	*tmexC3*	*tmexD3*	*toprJ1*	2018	N/A	USA
Pseudomonas aeruginosa	DAFSGK000000000	13ARS_VSM0626	*tmexC3*	*tmexD3*	*toprJ1*	2018	N/A	USA
Pseudomonas aeruginosa	DAFSDZ000000000	14ARS-VSM1187	*tmexC*	*tmexD3*	*toprJ*	2018	N/A	USA
Pseudomonas aeruginosa	DAFSHA000000000	13ARS_VSM0040	*tmexC3*	*tmexD3*	*toprJ1*	2018	N/A	USA
Pseudomonas aeruginosa	DAFSGN000000000	13ARS_VSM0188	*tmexC3*	*tmexD3*	*toprJ1*	2018	N/A	USA
Pseudomonas aeruginosa	DAFSGM000000000	13ARS_VSM0193	*tmexC3*	*tmexD3*	*toprJ1*	2018	N/A	USA
Pseudomonas aeruginosa	DAFSGL000000000	13ARS_VSM0192	*tmexC3*	*tmexD3*	*toprJ1*	2018	N/A	USA
Pseudomonas aeruginosa	JTXA00000000	AZPAE14705	*tmexC3*	*tmexD3*	*toprJ1*	2012	Homo sapiens	Greece
Pseudomonas aeruginosa	JTXC00000000	AZPAE14703	*tmexC3*	*tmexD3*	*toprJ1*	2012	Homo sapiens	Philippines
Pseudomonas aeruginosa	DAFSFZ000000000	14ARS-CVM0079	*tmexC3*	*tmexD3*	*toprJ1*	2018	N/A	USA
Pseudomonas aeruginosa	DAFSHZ000000000	13ARS_CVM0081	*tmexC3*	*tmexD3*	*toprJ1*	2018	N/A	USA
Pseudomonas aeruginosa	DAFSFY000000000	14ARS-CVM0005	*tmexC3*	*tmexD3*	*toprJ1*	2018	N/A	USA
Pseudomonas aeruginosa	DAFSGJ000000000	13ARS_VSM0340	*tmexC3*	*tmexD3*	*toprJ1*	2018	N/A	USA
Pseudomonas aeruginosa	DAFSGP000000000	13ARS_VSM0038	*tmexC3*	*tmexD3*	*toprJ1*	2018	N/A	USA
Pseudomonas aeruginosa	DAFSFB000000000	13ARS_VSM0339	*tmexC3*	*tmexD3*	*toprJ1*	2018	N/A	USA
Pseudomonas aeruginosa	DAFSGO000000000	13ARS_VSM0046	*tmexC3*	*tmexD3*	*toprJ1*	2018	N/A	USA
Pseudomonas aeruginosa	DAFSEO000000000	14ARS-NKI0252	*tmexC*	*tmexD3*	*toprJ*	2018	N/A	USA
Pseudomonas aeruginosa	DAFSGR000000000	13ARS_VSM0344	*tmexC3*	*tmexD3*	*toprJ1*	2018	N/A	USA
Pseudomonas aeruginosa	DAFSEA000000000	14ARS-VSM1054	*tmexC*	*tmexD3*	*toprJ*	2018	N/A	USA
Pseudomonas aeruginosa	DAFSHS000000000	13ARS_GMH0080	*tmexC3*	*tmexD3*	*toprJ1*	2018	N/A	USA
Pseudomonas aeruginosa	DAFSHT000000000	13ARS_GMH0171	*tmexC3*	*tmexD3*	*toprJ1*	2018	N/A	USA
Pseudomonas aeruginosa	DAFSHR000000000	13ARS_GMH0172	*tmexC3*	*tmexD3*	*toprJ1*	2018	N/A	USA
Pseudomonas aeruginosa	DAFSDV000000000	14ARS-VSM0988	*tmexC*	*tmexD3*	*toprJ*	2018	N/A	USA
Pseudomonas aeruginosa	ABHICR000000000	2022JQ-00128	*tmexC3*	*tmexD3*	*toprJ1*	2022	Homo sapiens	USA
Pseudomonas aeruginosa	ABHIBL000000000	2022JQ-00103	*tmexC3*	*tmexD3*	*toprJ1*	2022	Homo sapiens	USA
Pseudomonas aeruginosa	DAFSGY000000000	13ARS_STU0024	*tmexC3*	*tmexD3*	*toprJ1*	2018	N/A	USA
Pseudomonas aeruginosa	DAFSGV000000000	13ARS_STU0044	*tmexC3*	*tmexD3*	*toprJ1*	2018	N/A	USA
Pseudomonas aeruginosa	DAFSGE000000000	13ARS_VSM0732	*tmexC3*	*tmexD3*	*toprJ1*	2018	N/A	USA
Pseudomonas aeruginosa	DAFSED000000000	14ARS-VSM0987	*tmexC*	*tmexD3*	*toprJ*	2018	N/A	USA
Pseudomonas aeruginosa	DAFSEB000000000	14ARS-VSM0876	*tmexC*	*tmexD3*	*toprJ*	2018	N/A	USA
Pseudomonas aeruginosa	DAFSGH000000000	13ARS_VSM0743	*tmexC3*	*tmexD3*	*toprJ1*	2018	N/A	USA
Pseudomonas aeruginosa	DAFSFD000000000	14ARS-VSM0866	*tmexC3*	*tmexD3*	*toprJ1*	2018	NA	USA

Pseudomonas aeruginosa	DAFSEG000000000	14ARS-VSM0870	*tmexC*	*tmexD3*	*toprJ*	2018	N/A	USA
Pseudomonas aeruginosa	DAFSEZ000000000	13ARS_VSM0736	*tmexC3*	*tmexD3*	*toprJ*	2018	N/A	USA
Pseudomonas aeruginosa	DAFSGQ000000000	13ARS_VSM1021	*tmexC3*	*tmexD3*	*toprJ1*	2018	N/A	USA
Pseudomonas aeruginosa	DAFSGT000000000	13ARS_VSM0820	*tmexC3*	*tmexD3*	*toprJ1*	2018	N/A	USA
Pseudomonas aeruginosa	DAFSGG000000000	13ARS_VSM0740	*tmexC3*	*tmexD3*	*toprJ1*	2018	N/A	USA
Pseudomonas aeruginosa	DAFSGD000000000	13ARS_VSM0738	*tmexC3*	*tmexD3*	*toprJ1*	2018	N/A	USA
Pseudomonas putida	CAHPRN000000000	BB1451	*tmexC*	*tmexD2*	*toprJ1*	2017	environment	Ghana
Pseudomonas putida	CAHPSD000000000	BB1456	*tmexC*	*tmexD2*	*toprJ1*	2017	environment	Ghana
Pseudomonas putida	CAHPRR000000000	BB1483	*tmexC*	*tmexD2*	*toprJ1*	2017	environment	Ghana
Pseudomonas putida	LR813085	BB1456_hybrid	*tmexC*	*tmexD*	*toprJ*	2017	environments	Ghana
Pseudomonas putida	LR813083	BB1451_hybrid	*tmexC*	*tmexD*	*toprJ*	2017	environments	Ghana
Pseudomonas putida	CAHPSA000000000	BB1452	*tmexC*	*tmexD2*	*toprJ1*	2020	environment	Ghana
Pseudomonas putida	CP062218	SY153	*tmexC*	*tmexD3*	*toprJ*	2012	Homo sapiens	China
Pseudomonas putida	JAJNEE000000000	991_21	*tmexC2*	*tmexD3*	*toprJ1*	2021	Homo sapiens	Brazil
Pseudomonas putida	JALKHN000000000	T1647	*tmexC1*	*tmexD3*	*toprJ1*	2016	Homo sapiens	China
Pseudomonas putida	CAHPRN000000000	BB1451-2	*tmexC2*	*tmexD2*	*toprJ1*	2020	environmental_	Ghana
Pseudomonas putida	CAHPSD000000000	BB1456-2	*tmexC2*	*tmexD2*	*toprJ1*	2020	environmental_	Ghana
Pseudomonas putida	CP143524-CP143525	MT178	*tmexC2*	*tmexD3*	*toprJ1*	2023	environmental_	China
Pseudomonas putida	KY883660	SY153-1	*tmexC1*	*tmexD1*	*toprJ1*	2012	Homo sapiens	China
Pseudomonas putida	KU130294	12969	*tmexC*	*tmexD2*	*toprJ1*	2015	Homo sapiens	China
Pseudomonas putida	CP017073	PP112420	*tmexC1*	*tmexD1*	*toprJ1*	2011	Homo sapiens	China
Pseudomonas putida	CP061722-CP061724	ZXPA-20	*tmexC2*	*tmexD3*	*toprJ*	2019	animals	China
Pseudomonas putida	JAMHFX000000000	DS2	*tmexC*	*tmexD*	*toprJ1*	2018	enviroment	China
Pseudomonas putida	CP134602-CP134603	L2890	*tmexC*	*tmexD2*	*toprJ1*	2020	Homo sapiens	China
Pseudomonas putida	JALKHB000000000	T815 2	*tmexC1*	*tmexD3*	*toprJ1*	2016	Homo sapiens	China
Pseudomonas putida	JALPNP000000000	T901	*tmexC1*	*tmexD2*	*toprJ1*	2020	Homo sapiens	China
Pseudomonas putida	JALKHK000000000	T113-2	*tmexC1*	*tmexD2*	*toprJ1*	2020	Homo sapiens	China
Pseudomonas putida	JALKHJ000000000	T831	*tmexC1*	*tmexD2*	*toprJ1*	2020	Homo sapiens	China
Pseudomonas putida	CP120969	AHSWHJXPP1	*tmexC*	*tmexD*	*toprJ1*	2014	Homo sapiens	China
Pseudomonas putida	JALKHH000000000	T844	*tmexC1*	*tmexD2*	*toprJ1*	2020	Homo sapiens	China
Pseudomonas putida	JALKHG000000000	T883	*tmexC1*	*tmexD2*	*toprJ1*	2020	Homo sapiens	China
Pseudomonas putida	JALKHL000000000	T1006	*tmexC1*	*tmexD2*	*toprJ1*	2020	Homo sapiens	China
Pseudomonas putida	JALKHI000000000	T84-2	*tmexC1*	*tmexD2*	*toprJ1*	2020	Homo sapiens	China
Pseudomonas juntendi	JASGOH000000000	PP-E68B	*tmexC3*	*tmexD3*	*toprJ1*	2015	Homo sapiens	Pakistan
Pseudomonas juntendi	JAKGAU000000000	AR 06_Mez(R)_Tob(R	*tmexC*	*tmexD1*	*toprJ1*	2019	environmental	India
Pseudomonas juntendi	JAOCFX000000000	GD03792	*tmexC3*	*tmexD3*	*toprJ1*	2018	Homo sapiens	Pakistan
Pseudomonas juntendi	JAOCBV000000000	GD03901	*tmexC3*	*tmexD3*	*toprJ1*	2018	Homo sapiens	Pakistan
Pseudomonas juntendi	JALKHF000000000	F113	*tmexC1*	*tmexD1*	*toprJ1*	2018	Homo sapiens	China
Pseudomonas juntendi	JACGDD000000000	10618	*tmexC3*	*tmexD3*	*toprJ1*	2010	Homo sapiens	Brazil
Pseudomonas juntendi	JACGDA000000000	11213	*tmexC3*	*tmexD3*	*toprJ1*	2010	Homo sapiens	Brazil
Pseudomonas juntendi	JALLLY000000000	CCBH26530	*tmexC3*	*tmexD3*	*toprJ1*	2019	Homo sapiens	Brazil
Pseudomonas juntendi	JALLLV000000000	CCBH28261	*tmexC3*	*tmexD3*	*toprJ1*	2020	Homo sapiens	Brazil
Pseudomonas juntendi	CP118678-CP118680	GDW21C697WI	*tmexC*	*tmexD1*	*toprJ*	2020	animals	china
Pseudomonas juntendi	CP079903	K37-3	*tmexC1*	*tmexD3*	*toprJ1*	2020	Homo sapiens	China
Pseudomonas juntendi	JAJTOR000000000	psp210002463	*tmexC*	*tmexD*	*toprJ1*	2021	Homo sapiens	China
Pseudomonas juntendi	CP091088-CP091090	PP_2463	*tmexC*	*tmexD*	*toprJ1*	2021	Homo sapiens	China
Pseudomonas chengduensis	AVQF00000000	EGD-AQ5	*tmexC*	*tmexD*	*toprJ*	2012	environment	India
Pseudomonas chengduensis	LLXP00000000	ED1	*tmexC*	*tmexD*	*toprJ*	2009	environments	Argentina
Pseudomonas chengduensis	JAOCHG000000000	GD03756	*tmexC*	*tmexD*	*toprJ*	2018	Homo sapiens	Pakistan
Pseudomonas chengduensis	CP095766	T1624	*tmexC1*	*tmexD1*	*toprJ1*	2020	Homo sapiens	China
Pseudomonas chengduensis	JACFYZ000000000	402	*tmexC*	*tmexD*	*toprJ*	2018	environmental	France
Pseudomonas chengduensis	CP129400	WD211	*tmexC*	*tmexD*	*toprJ*	2022	environment	China
Pseudomonas chengduensis	JAOCIC000000000	GD03734	*tmexC*	*tmexD*	*toprJ*	2018	Homo sapiens	Pakistan
Pseudomonas chengduensis	ANGB00000000	34 IA	*tmexC*	*tmexD*	*toprJ*	2006	environment	Italy
Pseudomonas chengduensis	JAOCHO000000000	GD03748	*tmexC*	*tmexD*	*toprJ*	2018	Homo sapiens	Pakistan
Pseudomonas chengduensis	JAOCBD000000000	GD03921	*tmexC*	*tmexD*	*toprJ2*	2018	Homo sapiens	Pakistan
Pseudomonas psychrotolerans	FMWB00000000	DSM 15758	*TMexC3*	*TMexD3*	*TOprJ1*	2002	Animals	Austria
Pseudomonas sp.	QLYW00000000	MDMC17	*TMexC*	*TMexD*	*TOprJ*	2002	environments	Morocco

Pseudomonas sp.	QLYV00000000	MDMC216	*TMexC*	*TMexD*	*TOprJ*	2002	environments	Morocco
Pseudomonas sp.	QLYU00000000	MDMC224	*TMexC*	*TMexD*	*TOprJ*	2002	environments	Morocco
Pseudomonas sp.	CP031606	phDV1	*TMexC*	*TMexD*	*TOprJ*	2002	environment	Germany
Pseudomonas toyotomiensis	NIQV00000000	DSM 26169	*TMexC*	*TMexD*	*TOprJ*	2010	environment	Japan
Pseudomonas sp.	CP097103-CP097105	NY11382	*TMexC3*	*TMexD3*	*TOprJ1*	2011	Homo sapiens	China
Pseudomonas kurunegalensis	OR178467	NMI3456/12	*TMexC*	*TMexD*	*TOprJ*	2012	Homo sapiens	Poland
Pseudomonas kurunegalensis	CP128548	NMI3456_12	*TMexC*	*TMexD*	*TOprJ*	2012	Homo sapiens	Poland
Pseudomonas sp. LPH1	CP017290	LPH1	*TMexC*	*TMexD*	*TOprJ*	2012	Homo sapiens	USA
Pseudomonas kurunegalensis	JAJSRD000000000	NMI3044_13	*TMexC*	*TMexD*	*TOprJ*	2013	Homo sapiens	Poland
Pseudomonas sp.	PGZI00000000	NA	*TMexC*	*TMexD*	*TOprJ*	2013	environmental	Japan
Pseudomonas sp.	CP045553	13159349	*TMexC6*	*TMexD6*	*TOprJ1b*	2013	Homo sapiens	China
Pseudomonas sp.	LMMB00000000	Leaf83	*TMexC*	*TMexD*	*TOprJ*	2013	environment	Switzerland
Pseudomonas arcuscaelestis	JACOPV000000000	P66	*TMexC3*	*TMexD3*	*TOprJ1*	2014	animals	Turkey
Pseudomonas guariconensis	PJCQ00000000	MR144	*TMexC3*	*TMexD3*	*TOprJ1*	2014	environments	Nigeria
Pseudomonas guariconensis	PJCR00000000	MR149	*TMexC3*	*TMexD3*	*TOprJ1*	2014	environments	Nigeria
Pseudomonas guariconensis	PJCP00000000	MR119	*TMexC3*	*TMexD3*	*TOprJ1*	2014	environments	Nigeria
Pseudomonas oleovorans	JTFL00000000	S1	*TMexC*	*TMexD*	*TOprJ*	2014	N/A	China
Pseudomonas sp.	CP045554	NY5710	*TMexC1*	*TMexD1*	*TOprJ1*	2014	Homo sapiens	China
Pseudomonas sp.	JBOY00000000	RL	*TMexC*	*TMexD*	*TOprJ1*	2014	N/A	India
Pseudomonas sp.	PGLR00000000	ZH-FAD	*TMexC*	*TMexD*	*TOprJ*	2014	environments	China
Pseudomonas toyotomiensis	BBQO00000000	KF710	*TMexC*	*TMexD*	*TOprJ*	2014	environment	Japan
Pseudomonas monteilii	BCAP00000000	GTC 10899	*TMexC3*	*TMexD3*	*TOprJ1*	2015	NA	Japan
Pseudomonas sp.	SCGA00000000	ALS1279	*TMexC*	*TMexD*	*TOprJ*	2015	enviroment	USA
Pseudomonas sp.	CP041933	BJP69	*TMexC6*	*TMexD6*	*TOprJ1b*	2015	Homo sapiens	China
Pseudomonas sp.	ML178807	HS-2	*TMexC*	*TMexD*	*TOprJ*	2015	enviroment	China
Pseudomonas sp.	JAJSRO000000000	NMI542_15	*TMexC3*	*TMexD3*	*TOprJ1*	2015	Homo sapiens	Poland
Pseudomonas alcaliphila	FNAE00000000	JCM 10630	*TMexC*	*TMexD*	*TOprJ*	2016	N/A	USA
Pseudomonas asiatica	SWEL00000000	MY34	*TMexC3*	*TMexD3*	*TOprJ1*	2016	Homo sapiens	Myanmar
Pseudomonas kurunegalensis	JALKHD000000000	T2904	*TMexC1*	*TMexD2*	*TOprJ1*	2016	Homo sapiens	China
Pseudomonas kurunegalensis	JALKHE000000000	T2909-1	*TMexC1*	*TMexD2*	*TOprJ1*	2016	Homo sapiens	China
Pseudomonas kurunegalensis	JALKHM000000000	T103	*TMexC1*	*TMexD2*	*TOprJ1*	2016	Homo sapiens	China
Pseudomonas kurunegalensis	JALKHO000000000	T363	*TMexC*	*TMexD2*	*TOprJ1*	2016	Homo sapiens	CHINA
Pseudomonas kurunegalensis	JALPNR000000000	T362	*TMexC*	*TMexD2*	*TOprJ1*	2016	Homo sapiens	CHINA
Pseudomonas mendocina	JASGRV000000000	BC-E258	*TMexC3*	*TMexD3*	*TOprJ1*	2016	Homo sapiens	Bangladesh
Pseudomonas oleovorans	JALKGU000000000	T113	*TMexC3*	*TMexD3*	*TOprJ1*	2016	Homo sapiens	China
Pseudomonas oleovorans	RHRS00000000	PO_271	*TMexC*	*TMexD2*	*TOprJ1*	2016	Homo sapiens	Pakistan
Pseudomonas sihuiensis	LT629797	KCTC 32246	*TMexC*	*TMexD*	*TOprJ*	2016	N/A	USA
Pseudomonas sp.	QYYB00000000	KB-10	*TMexC*	*TMexD*	*TOprJ*	2016	enviroment	Saudi Arabia
Pseudomonas sp.	FPJD00000000	NFACC19-2	*TMexC*	*TMexD*	*TOprJ*	2016	N/A	USA
Pseudomonas sp.	FMXG00000000	NFPP33	*TMexC*	*TMexD*	*TOprJ*	2016	N/A	USA
Pseudomonas sp.	RHQP00000000	297	*TMexC*	*TMexD*	*TOprJ*	2016	Homo sapiens	Pakistan
Pseudomonas stutzeri	RHRM00000000	NA	*Temxc3*	*Tmexd3*	*Toprj1b*	2016	environment	Pakistan
Pseudomonas stutzeri	RHQZ00000000	PS_234	*TMexC6*	*TMexD6*	*TOprJ1b*	2016	environment	Pakistan
Pseudomonas asiatica	SWEH00000000	MY601	*TMexC*	*TMexD*	*TOprJ*	2017	Homo sapiens	Myanmar
Pseudomonas monteilii	CP043395	170918607	*TMexC1*	*TMexD1*	*TOprJ1*	2017	Homo sapiens	China
Pseudomonas monteilii	CP043396	170620603RE	*TMexC6*	*TMexD6*	*TOprJ1b*	2017	Homo sapiens	China
Pseudomonas sp.	CP024478	HLS-6	*TMexC1*	*TMexD1*	*TOprJ1*	2017	environment	China
Pseudomonas sp.	DJSX00000000	UBA6323	*MexC*	*TMexD*	*TOprJ*	2017	environmental	USA
Pseudomonas sp.	DLMS00000000	UBA7530	*TMexC*	*TMexD*	*TOprJ*	2017	environmental	USA
Pseudomonas chaetocerotis	JACFYX000000000	536 4	*TMexC*	*TMexD*	*TOprJ*	2018	environment	France
Pseudomonas mendocina	JADTQG000000000	PSB00032	*TMexC*	*TMexD*	*TOprJ*	2018	Homo sapiens	USA
Pseudomonas oleovorans	JAOCJE000000000	GD03704	*TMexC*	*TMexD2*	*TOprJ1*	2018	environmental	Pakistan
Pseudomonas oleovorans	JAOCJD000000000	GD03705	*TMexC*	*TMexD2*	*TOprJ1*	2018	environmental	Pakistan
Pseudomonas oleovorans	CP104579	GD04132	*TMexC*	*TMexD2*	*TOprJ1*	2018	Homo sapiens	Pakistan
Pseudomonas shirazica	CP063456-CP063457	A28	*TMexC3*	*TMexD3*	*TOprJ1*	2018	environment	China
Pseudomonas sp.	DMTR00000000	UBA9381	*TMexC*	*TMexD2*	*TOprJ1*	2018	environmental	Australia
Pseudomonas sp.	DNBU00000000	UBA9370	*TMexC*	*TMexD*	*TOprJ1*	2018	environmental	Australia
Pseudomonas sp.	JAMSKE000000000	18082574	*TMexC3*	*TMexD3*	*TOprJ1*	2018	Homo sapiens	China

Pseudomonas sp.	JAMSJX000000000	18101001-2	*TMexC3*	*TMexD3*	*TOprJ1*	2018	Homo sapiens	China
Pseudomonas sp.	JAMSKB000000000	18102011	*TMexC3*	*TMexD3*	*TOprJ1*	2018	Homo sapiens	China
Pseudomonas sp.	CP051625	gcc21	*TMexC1*	*TMexD1*	*TOprJ1*	2018	Homo sapiens	China
Pseudomonas sp.	JAOCJM000000000	GD03696	*TMexC3*	*TMexD3*	*TOprJ1*	2018	Homo sapiens	Pakistan
Pseudomonas sp.	CP104583	GD03721	*TMexC*	*TMexD2*	*TOprJ1*	2018	environment	Pakistan
Pseudomonas sp.	JAOCIO000000000	GD03722	*TMexC*	*TMexD2*	*TOprJ1*	2018	environmental	Pakistan
Pseudomonas sp.	JAOCLT000000000	GD03909	*TMexC*	*TMexD2*	*TOprJ1*	2018	Homo sapiens	Pakistan
Pseudomonas sp.	CP104582	GD03919	*TMexC*	*TMexD*	*TOprJ1*	2018	Homo sapiens	Pakistan
Pseudomonas sp.	JACETQ000000000	MCMED-G46	*TMexC*	*TMexD*	*TOprJ*	2018	environmental_	Spain
Pseudomonas fulva	CP064943-CP064945	ZDHY316	*TMexC1*	*TMexD1*	*TOprJ1*	2019	Homo sapiens	China
Pseudomonas fulva	CP064946-CP064948	ZDHY414	*TMexC1*	*TMexD1*	*TOprJ1*	2019	Homo sapiens	China
Pseudomonas hunanensis	CP079827-CP079828	HD6515	*TMexC3*	*TMexD3*	*TOprJ1*	2019	Homo sapiens	China
Pseudomonas monteilii	JAHWIU000000000	HD6516	*TMexC3*	*TMexD3*	*TOprJ1*	2019	environmental	China
Pseudomonas monteilii	JAFMZC000000000	ZDHY373	*TMexC3*	*TMexD3*	*TOprJ1*	2019	Homo sapiens	China
Pseudomonas monteilii	JAFMZB000000000	ZDHY354	*TMexC3*	*TMexD3*	*TOprJ*	2019	Homo sapiens	China
Pseudomonas qingdaonensis	CP129981	L1712hy	*TMexC*	*TMexD*	*TOprJ*	2019	Homo sapiens	China
Pseudomonas sp.	JACANC000000000	165	*TMexC*	*TMexD*	*TOprJ*	2019	Animals	China
Pseudomonas sp.	LR733263	Pseudomonas sp. 8O	*TMexC*	*TMexD*	*TOprJ*	2019	enviroment	Portugal
Pseudomonas sp.	JAHWIX000000000	HD6421	*TMexC3*	*TMexD3*	*TOprJ3*	2019	environmental	China
Pseudomonas sp.	JAHWIW000000000	HD6422	*TMexC3*	*TMexD3*	*TOprJ3*	2019	environmental	China
Pseudomonas stutzeri	CP063358-CP063359	ZDHY95	*TMexC6*	*TMexD6*	*TOprJ1b*	2019	Homo sapiens	China
Pseudomonas sp.	CABHPC000000000	udomonas_sp_nbed6b	*TMexC*	*TMexD2*	*TOprJ*	2019	environmental	Netherlands
Pseudomonas alcaligenes	BPMN00000000	KAM428	*TMexC*	*TMexD2*	*TOprJ1*	2020	NA	Japan
Pseudomonas alcaligenes	BPMO00000000	KAM429	*TMexC*	*TMexD2*	*TOprJ1*	2020	NA	Japan
Pseudomonas alcaligenes	BPMP00000000	KAM430	*TMexC*	*TMexD2*	*TOprJ1*	2020	NA	Japan
Pseudomonas alcaligenes	BPMQ00000000	KAM432	*TMexC*	*TMexD2*	*TOprJ1*	2020	NA	Japan
Pseudomonas alcaligenes	BPMR00000000	KAM434	*TMexC*	*TMexD2*	*TOprJ1*	2020	NA	Japan
Pseudomonas alcaligenes	BPMS00000000	KAM435	*TMexC*	*TMexD2*	*TOprJ1*	2020	NA	Japan
Pseudomonas alcaligenes	BPMT00000000	KAM436	*TMexC*	*TMexD2*	*TOprJ1*	2020	NA	Japan
Pseudomonas guariconensis	JALPNQ000000000	T305	*TMexC1*	*TMexD2*	*TOprJ1*	2020	Homo sapiens	China
Pseudomonas sp.	JAAQXB000000000	WS 5019	*TMexC*	*TMexD*	*TOprJ*	2020	environmental	Germany
Pseudomonas yangonensis	BLJV00000000	MY63	*TMexC*	*TMexD*	*TOprJ*	2020	Homo sapiens	Japan
Pseudomonas yangonensis	BLJW00000000	MY101	*TMexC*	*TMexD*	*TOprJ*	2020	Homo sapiens	Japan
Pseudomonas yangonensis	BLJU00000000	MY50	*TMexC*	*TMexD*	*TOprJ*	2020	NA	Japan
Pseudomonas sp.	DXBL00000000	CHK174-787	*TMexC3*	*TMexD3*	*TOprJ1*	2021	animals	UK
Pseudomonas sp.	CP113226-CP113227	T75	*TMexC*	*TMexD3*	*TOprJ*	2021	animals	China
Pseudomonas sp.	JAQCOA000000000	PRO1	*TMexC*	*TMexD2*	*TOprJ1*	2021	environmental	China
Pseudomonas alcaligenes	AP024354	KAM426	*TMexC6*	*TMexD6*	*TOprJ1b*	2021	Homo sapiens	Japan
Pseudomonas plecoglossicida	JAVHYJ000000000	P15	*TMexC*	*TMexD2*	*TOprJ1*	2021	Homo sapiens	Egypt
Pseudomonas sp.	CP104044	SD17-1	*TMexC*	*TMexD*	*TOprJ1*	2021	Homo sapiens	China
Pseudomonas toyotomiensis	Cp070505	SM2	*TMexC*	*TMexD*	*TOprJ*	2021	environment	ted arab emir
Pseudomonas mendocina	CP115817	GD22SC3150TT	*TMexC6*	*TMexD6*	*TOprJ1b*	2022	environment	china
Pseudomonas sp.	CALNWT000000000	Marseille-Q0931	*TMexC*	*TMexD*	*TOprJ*	2022	Homo sapiens	France
Pseudomonas sp.	CP113432	ZM23	*TMexC*	*TMexD2*	*TOprJ1*	2022	Homo sapiens	china
Pseudomonas sp.	JANCLO000000000	ZM24	*TMexC*	*TMexD2*	*TOprJ1*	2022	environmental	China
Pseudomonas sp.	JANCLP000000000	ZM25	*TMexC*	*TMexD2*	*TOprJ1*	2022	environmental	China
Pseudomonas alcaliphila	JAVDVG000000000	3512	*TMexC*	*TMexD*	*TOprJ*	2023	NA	USA
Pseudomonas guariconensis	JAUDEX000000000	F8_WW	*TMexC*	*TMexD2*	*TOprJ1*	2023	animals	Thailand
Pseudomonas guariconensis	JAUDEY000000000	F15_WW	*TMexC*	*TMexD2*	*TOprJ1*	2023	environment	Thailand
Pseudomonas guariconensis	JAUDEZ000000000	F17-WW	*TMexC*	*TMexD2*	*TOprJ1*	2023	environment	Thailand
Pseudomonas sp.	JAUSRJ000000000	3400	*TMexC*	*TMexD*	*TOprJ*	2023	NA	USA
